# Association between multimodal pelvic floor rehabilitation and symptom improvement in postpartum women with low back pain and constipation: a retrospective cohort study

**DOI:** 10.3389/fmed.2026.1909450

**Published:** 2026-10-27

**Authors:** Yegai Feng, Guihua Xiao, Yonggang Zhu, Yongxue Li, Xueliang Xu

**Affiliations:** 1 Comprehensive Rehabilitation Department, The First People’s Hospital of Lianyungang, Lianyungang, Jiangsu, China; 2 Lianyungang Hospital of Traditional Chinese Medicine Affiliated to Nanjing University of Chinese Medicine, Lianyungang, Jiangsu, China

**Keywords:** biofeedback, constipation, core stability training, low back pain, low-frequency electrical stimulation, pelvic floor rehabilitation, postpartum rehabilitation, propensity score matching

## Abstract

**Objective:**

To examine the association between multimodal pelvic floor rehabilitation and improvements in low back pain, functional disability, and constipation-related symptoms among postpartum women with concomitant low back pain and constipation.

**Methods:**

This single-center retrospective observational cohort study screened 410 postpartum women who underwent rehabilitation assessment between January 2023 and December 2025. Of these, 352 met the baseline eligibility criteria and initiated rehabilitation, including 170 women who initiated conventional active rehabilitation (active comparator) and 182 who initiated multimodal pelvic floor rehabilitation. Treatment initiation was defined as cohort entry, irrespective of subsequent treatment completion or availability of follow-up outcome data. Evaluable follow-up outcomes were available for 300 of 352 treatment initiators (85.2%), with a mean observed baseline-to-follow-up interval of approximately 57.6 days; 52 participants (14.8%) had no evaluable follow-up assessment. Primary baseline-adjusted models incorporated the corresponding baseline outcome, the observed baseline-to-follow-up interval, and clinically selected baseline covariates. Inverse-probability-of-follow-up weighting (IPCW) and a 56-day landmark-anchored delta sensitivity analysis were performed to assess the potential influence of differential follow-up and departures from the missing-at-random assumption.

**Results:**

In the primary observed-follow-up analysis, multimodal rehabilitation was associated with lower follow-up NRS score (adjusted *β* = −1.58, 95% CI: −1.79 to −1.37), lower ODI score (*β* = −9.71, 95% CI: −10.65 to −8.78), lower PAC-SYM score (*β* = −0.62, 95% CI: −0.69 to −0.54), and higher weekly bowel-movement frequency (*β* = 1.54, 95% CI: 1.38–1.69); all *P* < 0.001. IPCW-weighted analyses yielded materially similar estimates, and the associations remained evident under the severe landmark-anchored MNAR sensitivity scenario.

**Conclusion:**

In this retrospective observational cohort, multimodal pelvic floor rehabilitation was associated with greater improvements in low back pain, functional disability, and constipation-related symptoms than the conventional active rehabilitation comparator. However, the multimodal strategy involved greater treatment exposure, including more supervised treatment sessions and professional contact. Therefore, part of the observed strategy-level association may be attributable to differences in treatment exposure or intensity rather than to the specific multimodal components alone. Given the observational design, these findings should be interpreted as associations rather than evidence of a causal or component-specific treatment effect.

## 1 Introduction

Postpartum low back pain is one of the common discomfort symptoms during the puerperium and postpartum rehabilitation period. It may present as lumbosacral pain, restricted mobility, and symptom aggravation after prolonged standing or holding the infant, thereby affecting maternal daily activities, sleep quality, and childcare ability (1, 2). Meanwhile, constipation is also a common problem among postpartum women and may be associated with changes in pelvic floor support structures after pregnancy and childbirth, reduced abdominal pressure regulation, decreased physical activity, dietary changes, altered defecation habits, and psychological stress (3). Although low back pain and constipation are symptoms involving different systems, they often coexist in postpartum women and may jointly reflect reduced core stability, impaired coordination of the pelvic floor muscles, and abnormal regulation of abdominopelvic pressure (4).

The pelvic floor muscles are involved not only in urinary continence, fecal continence, and pelvic organ support, but also in maintaining lumbar-pelvic stability together with the transversus abdominis, multifidus, and diaphragm (5). Postpartum reductions in pelvic floor muscle strength, weakened muscular coordination, and difficulty in pelvic floor relaxation may increase the lumbosacral load, impair postural control, and affect the coordination between abdominal pressure generation and pelvic floor relaxation during defecation (6, 7). Therefore, interventions targeting pain alone or providing lifestyle guidance alone may be insufficient to simultaneously improve postpartum low back pain and constipation. In recent years, comprehensive rehabilitation interventions, including pelvic floor muscle training, biofeedback, low-frequency electrical stimulation, core stability training, and defecation behavior guidance, have been increasingly applied to postpartum pelvic floor functional recovery. Their advantages lie in multidimensional intervention targeting muscle strength recovery, neuromuscular control, abdominopelvic coordination, and reconstruction of defecation habits (8).

However, clinical evidence regarding the association between multimodal pelvic floor rehabilitation and concurrent improvement in postpartum low back pain and constipation-related symptoms remains limited, particularly in real-world clinical settings. Therefore, the present retrospective cohort study aimed to examine whether receipt of multimodal pelvic floor rehabilitation was associated with greater improvements in low back pain, functional disability, and constipation-related symptoms compared with the conventional active rehabilitation comparator. Multivariable regression, propensity score matching, and inverse probability of treatment weighting analyses were used to assess the robustness of the observed associations.

## 2 Study population and methods

### 2.1 Study design and study population

This study was designed as a single-center retrospective observational cohort study. Consecutive postpartum women who underwent baseline rehabilitation assessment and subsequently initiated postpartum rehabilitation treatment at the postpartum rehabilitation outpatient clinic or pelvic floor rehabilitation center of The First People's Hospital of Lianyungang between January 2023 and December 2025 were screened for eligibility.

For the present study, treatment initiation was used as the point of cohort entry. Participants were classified according to the rehabilitation strategy initiated at cohort entry as either the conventional active rehabilitation group (active comparator) or the multimodal pelvic floor rehabilitation group. Treatment completion and availability of follow-up outcome data were not used to determine study eligibility. The date of the first documented rehabilitation session was defined as the index date (time zero). The baseline assessment was defined as the most recent eligible rehabilitation assessment performed before or on the index date. The temporal sequence of baseline assessment, treatment initiation, final treatment session, and outcome assessment was reconstructed from the electronic medical record and rehabilitation information systems.

At baseline, postpartum constipation-related symptoms were defined using a study-defined symptom-based operational definition. Participants were required to have clinician-documented postpartum constipation together with at least two constipation-related symptom domains, including infrequent bowel movements, straining, hard or lumpy stools, sensation of incomplete evacuation, sensation of anorectal obstruction, or need for manual assistance with defecation. These symptom domains were selected with reference to the Rome IV symptom framework for functional constipation (9). However, because the required Rome IV chronicity criterion—symptoms fulfilled for the preceding 3 months with onset at least 6 months before diagnosis—could not be reliably established in this postpartum cohort, participants were not classified as having Rome IV-defined functional constipation.

The inclusion criteria were as follows: (1) age 18–45 years; (2) singleton pregnancy and completed delivery; (3) receipt of baseline postpartum rehabilitation assessment between 6 weeks and 12 months postpartum; (4) presence of postpartum low back pain with a baseline numerical rating scale (NRS) score ≥3; (5) concomitant constipation-related symptoms documented at baseline; and (6) initiation of either conventional or multimodal pelvic floor rehabilitation during the study period.

The exclusion criteria were as follows: (1) pre-pregnancy chronic low back pain, lumbar disc herniation, history of spinal surgery, spinal fracture, ankylosing spondylitis, or other definite spinal disorders; (2) inflammatory bowel disease, intestinal obstruction, intestinal tumors, severe hemorrhoidal disease, or other organic intestinal diseases; (3) severe postpartum hemorrhage, severe infection, malignant tumors, severe cardiac, hepatic, renal, or neurological disorders; and (4) pregnancy occurring again during the study period.

During the study period, 410 women were screened for eligibility. Fifty-eight women were excluded before treatment initiation because they did not meet the study-defined baseline eligibility criteria. The remaining 352 women initiated rehabilitation and constituted the treatment-initiation cohort, including 170 women who initiated conventional active rehabilitation comparator and 182 women who initiated multimodal pelvic floor rehabilitation. Treatment completion and follow-up availability were subsequently assessed as post-baseline characteristics rather than eligibility criteria.

This study was approved by the Ethics Committee of The First People's Hospital of Lianyungang on January 26, 2024 (Approval No. KY-20240115006-01). The Ethics Committee confirmed that the approval and waiver of informed consent covered the retrospective analysis of clinical records from the entire study period, January 2023 to December 2025, including records generated before the formal approval date. All data were anonymized before statistical analysis.

### 2.2 Treatment selection in routine clinical practice

Rehabilitation strategy was determined through shared clinical decision-making between the rehabilitation physician and the patient rather than random allocation or a formal treatment-assignment algorithm. Following baseline rehabilitation assessment, the physician evaluated symptom severity, pelvic floor muscle function, functional limitations, and rehabilitation needs and discussed the available rehabilitation strategies with the patient. Greater symptom burden or more evident pelvic floor dysfunction could favor recommendation of the multimodal rehabilitation program; however, no single symptom score, pelvic floor muscle-strength threshold, or other mandatory clinical criterion determined treatment allocation.

The final rehabilitation strategy reflected both the physician's clinical recommendation and the patient's preference and willingness to participate. Practical considerations, including treatment cost and insurance coverage, appointment availability, and access to the equipment required for device-assisted rehabilitation, could also influence the treatment strategy ultimately initiated. These factors represented routine real-world determinants of treatment choice rather than formal allocation criteria. Detailed socioeconomic circumstances, motivation, treatment expectations, and the relative contribution of individual practical considerations were not systematically captured as structured variables in the retrospective records.

Both rehabilitation strategies were provided within the same rehabilitation service. Rehabilitation assessment and treatment planning were undertaken by the same stable departmental rehabilitation physician team. Supervised treatment in both rehabilitation groups was delivered by the same departmental therapist team. Therapists delivering the multimodal program had additionally received specific training in pelvic floor rehabilitation and device-assisted treatment. The core clinical team, rehabilitation equipment, and institutional rehabilitation protocols did not undergo major changes during 2023–2025.

### 2.3 Rehabilitation protocols

Rehabilitation assessment and treatment planning were performed by rehabilitation physicians with at least 5 years of clinical experience in postpartum rehabilitation. Supervised pelvic floor rehabilitation was delivered by licensed rehabilitation therapists who had received standardized training in pelvic floor rehabilitation and had at least 3 years of relevant clinical experience. Treatment prescriptions were individualized according to baseline pelvic floor muscle strength, symptom severity, rehabilitation tolerance, and treatment progress.

#### 2.3.1 Conventional active rehabilitation (active comparator)

Participants in the conventional active rehabilitation group received routine postpartum health education, posture and activity guidance, dietary and lifestyle recommendations, and basic home-based pelvic floor muscle training (PFMT). PFMT consisted primarily of voluntary pelvic floor contraction and relaxation without biofeedback or electrical stimulation. Patients were instructed to perform three sets daily, with 10 slow contractions per set. Each slow contraction was maintained for approximately 5 s, followed by 5 s of relaxation, and each set was followed by 10 rapid contractions. Correct contraction technique was demonstrated during the initial outpatient visit, after which PFMT was performed predominantly at home without device-assisted feedback. Technique and progress were reviewed during scheduled outpatient reassessments, generally once weekly. Thus, this group represented an active rehabilitation comparator rather than a no-treatment control.

#### 2.3.2 Multimodal pelvic floor rehabilitation

Participants in the multimodal rehabilitation group received the same general postpartum health, posture, dietary, and lifestyle guidance as the conventional group; however, PFMT was delivered as a structured, therapist-supervised, and progressively adjusted program integrated with biofeedback, low-frequency electrical stimulation, core stabilization training, and constipation-related behavioral training.

**Supervised PFMT** consisted of three sets of 10 slow contractions, with each contraction maintained for 6–8 s followed by 6–8 s of relaxation, together with 10 rapid contractions after each set. Training intensity and contraction duration were progressively adjusted according to the participant's ability to correctly recruit and relax the pelvic floor muscles and according to biofeedback findings. Participants were additionally encouraged to perform two sets of the prescribed PFMT program at home each day.

Biofeedback training was performed using a PHENIX USB4 neuromuscular stimulation therapy device (ELECTRONIC CONCEPT LIGNON INNOVATION, 200 rue de Thor-Parc Eureka, 34000 Montpellier, France) equipped with a combined electrical stimulation and biofeedback intravaginal probe. The same device and intravaginal probe were used for both biofeedback and electrical stimulation, with the corresponding treatment prescription selected on the device according to the scheduled treatment component. During biofeedback training, the biofeedback prescription was selected, and participants performed repeated pelvic floor contraction-relaxation cycles while receiving real-time visual feedback on contraction amplitude and relaxation quality. Each biofeedback session lasted approximately 20 min and was generally performed twice weekly.

**Low-frequency electrical stimulation** was delivered using the same PHENIX USB4 device and the same combined electrical stimulation and biofeedback intravaginal probe described above, with the electrical stimulation prescription selected on the device. Stimulation was delivered at 35 Hz with a pulse width of 300 *μ*s and a duty cycle of 5 s of stimulation followed by 10 s of rest. Current intensity was gradually increased to the highest comfortably tolerated level that produced a perceptible pelvic floor contraction without pain, generally within 10–30 mA. Each electrical stimulation session lasted approximately 15 min and was generally performed twice weekly.

Core stabilization training included diaphragmatic breathing combined with transversus abdominis activation, maintenance of a neutral pelvic position, bridging exercises, and coordinated abdominal-pelvic floor activation. Each supervised session lasted approximately 20 min. Participants performed two sets of 10–12 repetitions for each exercise, with progression according to movement quality, pain tolerance, and lumbopelvic control.

Constipation-related behavioral training included regular toileting, appropriate defecation posture, avoidance of excessive straining, diaphragmatic breathing, pelvic floor relaxation during defecation, and individualized recommendations regarding dietary fiber and fluid intake. Participants were instructed to practice diaphragmatic breathing for approximately 5 min twice daily and to apply coordinated abdominal pressure generation and pelvic floor relaxation during defecation. Behavioral instructions were reinforced at each supervised visit.

In routine practice, conventional active rehabilitation comparator generally included approximately one outpatient reassessment per week together with daily home-based exercises, whereas the multimodal program was generally scheduled twice weekly for 4–8 weeks. Actual treatment exposure varied between participants and was therefore quantified using the number of sessions actually received and the interval between the first and final documented treatment sessions.

### 2.4 Data collection

Study data were collected from the hospital electronic medical record system, pelvic floor rehabilitation assessment system, and follow-up records. General demographic and clinical data included age, body mass index (BMI), postpartum time, mode of delivery, parity, neonatal birth weight, breastfeeding status, episiotomy, and degree of perineal laceration. Clinical symptom and functional data included baseline NRS pain score, Oswestry Disability Index (ODI) score, Patient Assessment of Constipation Symptoms (PAC-SYM) score, weekly bowel-movement frequency, Bristol Stool Form Scale score, Oxford pelvic floor muscle strength grade, Pelvic Floor Distress Inventory-20 (PFDI-20) score, duration of low back pain, duration of constipation, analgesic use, and laxative use. Validated Chinese-language versions of the ODI, PFDI-20, and PAC-SYM were administered (10–12). At baseline and follow-up, the questionnaires were administered during routine outpatient assessments by trained rehabilitation therapists. Participants generally completed the questionnaires independently. When a participant had difficulty understanding an item, the therapist provided standardized clarification without suggesting or influencing the response. Weekly bowel-movement frequency was determined from the participant's self-reported total number of bowel movements during the preceding 7 days and was recorded in the routine clinical assessment documentation. Oxford pelvic floor muscle strength was assessed by trained pelvic floor rehabilitation personnel using the modified Oxford grading scale. Because the assessments were conducted as part of routine clinical care, the assessors were aware of the rehabilitation strategy received, and independent blinded outcome assessment was not implemented. In some cases, the personnel conducting the outcome assessment may also have participated in rehabilitation delivery. Treatment-related data included rehabilitation modality, number of treatment sessions, and treatment adherence.

Treatment adherence was assessed from rehabilitation attendance records and documented home-training records. Clinic-based treatment adherence was defined as the proportion of prescribed rehabilitation sessions actually completed during the planned treatment period and was calculated as:

Treatment adherence (%) was calculated as the number of completed prescribed rehabilitation sessions divided by the number of prescribed rehabilitation sessions × 100%.

An adherence level ≥80% was considered good adherence for descriptive purposes. For home-based PFMT, adherence was based on patient-reported exercise completion documented during outpatient follow-up because home exercise was not electronically monitored. Treatment-related intolerance and adverse events documented in rehabilitation records or follow-up notes were also extracted. Adverse events were identified retrospectively from routine clinical documentation; no standardized prospective adverse-event surveillance protocol was used.

Treatment-course and follow-up information included treatment initiation status, treatment completion status, reason for treatment discontinuation where documented, availability of follow-up outcome assessment, and reason for missing follow-up where available. Participants who initiated rehabilitation but did not complete the planned treatment course or did not have a follow-up assessment remained part of the treatment-initiation cohort.

Temporal and treatment-exposure variables included the baseline assessment date, treatment initiation date, date of the final documented treatment session, outcome assessment date, actual number of treatment sessions received, and treatment adherence. The baseline-to-treatment initiation interval was calculated as the number of days between baseline assessment and treatment initiation. Actual treatment duration was calculated as the number of days between the first and final documented treatment sessions. The baseline-to-follow-up interval and treatment initiation-to-follow-up interval were calculated in days for participants with an evaluable follow-up assessment.

### 2.5 Outcome measures

Follow-up outcomes were obtained from the first complete post-initiation outcome assessment available during the routine rehabilitation follow-up period. The exact date of outcome assessment was recorded for each participant rather than assuming a uniform post-treatment assessment time point. Consequently, individual baseline-to-follow-up intervals were incorporated into the statistical analyses.

The co-primary continuous outcomes were follow-up NRS pain score and follow-up ODI score, representing pain intensity and pain-related functional disability, respectively. The corresponding baseline outcome value was included in each adjusted model. Higher NRS scores indicated greater pain severity, whereas higher ODI scores indicated greater functional limitation.

Follow-up PAC-SYM score and weekly bowel-movement frequency were treated as key secondary supportive continuous outcomes rather than additional confirmatory endpoints. Higher PAC-SYM scores indicated greater constipation-related symptom severity, whereas higher weekly bowel-movement frequency indicated more frequent bowel movements. Binary responder outcomes, alternative responder thresholds, and other sensitivity analyses were considered supportive or exploratory analyses.

For descriptive and sensitivity analyses, changes from baseline were additionally calculated. Changes in NRS, ODI, and PAC-SYM scores were calculated as the baseline value minus the follow-up value, whereas change in weekly defecation frequency was calculated as the follow-up value minus the baseline value. These change scores were not used as dependent variables in the primary continuous outcome models.

For binary supportive analyses of constipation-related symptom improvement, a clinically meaningful PAC-SYM response was defined as an absolute reduction of at least 0.6 points from baseline, based on the published minimal important difference for the PAC-SYM questionnaire (13, 14).

Because the validated PAC-SYM MID was derived predominantly from patients with chronic constipation rather than postpartum populations, this binary responder analysis was considered supportive rather than primary. A more conservative absolute reduction of ≥0.75 points and the originally used relative reduction of ≥30% were examined as alternative sensitivity thresholds.

Improvement in bowel-movement frequency was evaluated separately from PAC-SYM response. An increase of ≥2 bowel movements per week was retained only as an exploratory frequency-based endpoint and was not considered equivalent to the validated PAC-SYM MID. The two criteria were therefore no longer combined using an “OR*”* rule.

### 2.6 Statistical analysis

Statistical analyses were performed using R software (version 4.3.2). Continuous variables were expressed as the mean ± standard deviation or median (interquartile range), depending on their distribution, and categorical variables were expressed as frequencies and percentages. For comparisons of baseline characteristics between the two groups, continuous variables were analyzed using the independent-sample *t* test or Mann–Whitney *U*-test, and categorical variables were analyzed using the *χ*^2^ test or Fisher's exact test. Standardized mean differences (SMDs) were used to evaluate the balance of baseline variables between the two groups. A smaller SMD indicated better between-group balance, and an SMD <0.10 was generally considered to indicate good balance.

Outcome hierarchy and multiplicity. Follow-up NRS and ODI were treated as co-primary continuous outcomes. PAC-SYM and weekly bowel-movement frequency were considered key secondary supportive outcomes, while responder analyses, alternative response thresholds, propensity-score analyses, missing-data analyses, distribution-appropriate models, and subgroup analyses were supportive or sensitivity analyses. No prospectively registered statistical analysis plan with a formal multiplicity-adjustment or hierarchical testing procedure was available for this retrospective study. Therefore, no formal adjustment for multiplicity was applied, and reported *P values* should be interpreted as nominal. Statistical interpretation emphasized effect estimates and 95% confidence intervals, with the principal inference based on the co-primary NRS and ODI outcomes; secondary, supportive, and exploratory analyses were used to assess consistency and robustness rather than to establish additional confirmatory claims.

The treatment-initiation cohort comprised all 352 participants who initiated rehabilitation. Of these, 300 participants had an evaluable post-initiation outcome assessment and constituted the primary outcome-analysis cohort; the remaining 52 had no evaluable follow-up outcome assessment. The extent and pattern of unavailable follow-up outcomes were summarized according to rehabilitation group. Baseline characteristics were compared between participants with and without evaluable follow-up using standardized mean differences to assess the potential for differential follow-up.

Primary continuous-outcome analyses were conducted among participants with an observed follow-up assessment. Follow-up NRS, ODI, PAC-SYM, and weekly defecation frequency were modeled as dependent variables. Each model included rehabilitation strategy, the corresponding baseline outcome value, the observed baseline-to-follow-up interval in days, and clinically selected baseline covariates defined for the present analysis. Because an assessment time does not exist as an observed quantity for participants who never underwent follow-up, no hypothetical baseline-to-follow-up interval was assigned or imputed in the primary analysis.

To evaluate potential bias arising from differential follow-up availability, an inverse-probability-of-follow-up-weighted (IPCW) sensitivity analysis was performed. The denominator model estimated each participant's probability of having an evaluable follow-up assessment using logistic regression including rehabilitation strategy, age, BMI, postpartum time, mode of delivery, parity, baseline NRS score, baseline ODI score, baseline PAC-SYM score, baseline weekly defecation frequency, baseline Oxford pelvic floor muscle strength grade, baseline PFDI-20 score, analgesic use, and laxative use. The numerator model included rehabilitation strategy only. Stabilized inverse-probability-of-follow-up weights were calculated as the ratio of the predicted follow-up probability from the numerator model to that from the denominator model among participants with observed outcomes. To limit the influence of extreme weights, stabilized weights were truncated at the 1st and 99th percentiles. Weight diagnostics included the weight distribution, extreme weights, effective sample size, and standardized differences comparing the weighted outcome-evaluable sample with the full treatment-initiation cohort. Weighted outcome models used robust sandwich standard errors.

Reasons for unavailable follow-up were summarized descriptively but were not treated as baseline covariates because they were determined after cohort entry. Treatment completion, treatment duration, number of treatment sessions, and treatment adherence were likewise post-initiation variables and were not included in the primary IPCW model.

Because the missing-at-random (MAR) assumption could not be verified from the observed data, a *post hoc* landmark-anchored pattern-mixture sensitivity analysis was additionally performed. For the 52 participants without an evaluable follow-up assessment, the hypothetical assessment time was fixed at 56 days (8 weeks) after baseline rather than being imputed. This landmark provided a clinically interpretable common assessment time close to the approximately 57-day mean follow-up interval observed among participants with evaluable follow-up. No follow-up interval was imputed in this analysis.

Missing follow-up outcomes were imputed separately for NRS, ODI, PAC-SYM, and weekly bowel-movement frequency using predictive mean matching with 50 imputations. Each imputation model included rehabilitation strategy, age, BMI, postpartum time, mode of delivery, parity, baseline NRS score, baseline ODI score, baseline PAC-SYM score, baseline weekly bowel-movement frequency, baseline Oxford pelvic floor muscle strength grade, baseline PFDI-20 score, analgesic use, laxative use, and the landmark/observed assessment time. Treatment completion, actual number of treatment sessions, and treatment adherence were additionally included as auxiliary predictors to improve prediction of missing outcomes; these post-initiation variables were not included as adjustment covariates in the final outcome models. Documented reasons for unavailable follow-up were summarized descriptively but were not incorporated into the imputation model. Observed and MAR-imputed outcome distributions were compared descriptively by rehabilitation group.

To assess departures from MAR, a delta-adjustment pattern-mixture analysis progressively shifted the imputed outcomes of participants without follow-up in the multimodal rehabilitation group in a direction unfavorable to multimodal rehabilitation. Mild, moderate, and severe scenarios respectively added 0.5, 1.0, and 1.5 points to NRS; 3, 5, and 10 points to ODI; 0.15, 0.30, and 0.50 points to PAC-SYM; and subtracted 0.5, 1.0, and 1.5 bowel movements per week. Estimates were pooled across the 50 imputed datasets using Rubin's rules.

Assumptions of the linear regression models were assessed using graphical and numerical diagnostic procedures. Linearity and model specification were evaluated using residual-vs.-fitted plots. Homoscedasticity was assessed by examining residual-vs.-fitted plots and using the Breusch–Pagan test. Residual distribution was evaluated using normal quantile–quantile plots. Potentially influential observations were assessed using standardized residuals, leverage values, and Cook's distance. Observations with an absolute standardized residual >3, leverage greater than approximately 2p/n, or Cook's distance >4/n were flagged for further examination, where p denotes the number of estimated model parameters and n the model sample size. Sensitivity analyses excluding influential observations were performed when necessary to determine whether individual observations materially affected the estimated rehabilitation-group coefficient.

Because weekly bowel-movement frequency is a non-negative integer count outcome, a distribution-appropriate sensitivity analysis was performed using multivariable Poisson regression with a log link and robust sandwich standard errors. The model included rehabilitation strategy, baseline weekly bowel-movement frequency, the observed baseline-to-follow-up interval, and the same clinically selected baseline covariates used in the primary analysis. Overdispersion was assessed using the Pearson chi-square statistic divided by the residual degrees of freedom. Results were expressed as incidence rate ratios (IRRs) with 95% confidence intervals.

Because PAC-SYM is bounded on a 0–4 scale, a beta-regression sensitivity analysis was additionally performed. Follow-up PAC-SYM scores were rescaled to the open unit interval by dividing by 4. None of the observed follow-up PAC-SYM scores was exactly 0 or 4; therefore, no boundary correction was required. The beta-regression model used the same baseline adjustment set as the primary PAC-SYM model. For clinical interpretability, the rehabilitation-group contrast was standardized back to the original 0–4 PAC-SYM scale and reported as an adjusted average marginal difference with a 95% confidence interval.

Complete-case change-score analyses were performed as secondary sensitivity analyses. Among participants with evaluable baseline and follow-up assessments, changes in NRS, ODI, PAC-SYM, and weekly defecation frequency were calculated and analyzed using multivariable linear regression. Binary responder outcomes were also evaluated in the complete-case sample using multivariable logistic regression and were considered secondary supportive analyses. Because the binary responder endpoints were supportive and some responder cells were sparse, observed responder proportions and absolute between-group risk differences with 95% confidence intervals were emphasized as the primary descriptive measures for these endpoints. Adjusted odds ratios from conventional logistic regression were retained as secondary relative measures. As a sparse-data sensitivity analysis, Firth penalized logistic regression was additionally performed for each responder endpoint using the same clinically selected pre-treatment covariates as Sensitivity Model 1.

Sensitivity Model 1 was adjusted for clinically selected pre-treatment covariates, including age, BMI, postpartum time, mode of delivery, parity, baseline NRS pain score, baseline ODI score, baseline PAC-SYM score, baseline weekly defecation frequency, baseline Oxford pelvic floor muscle strength grade, analgesic use, and laxative use. Actual treatment duration, number of treatment sessions, and treatment adherence were considered post-initiation treatment-process variables rather than baseline confounders. Because these variables may themselves be influenced by the rehabilitation strategy initiated and may lie on the pathway between rehabilitation strategy and subsequent outcomes, they were not included in Sensitivity Model 1. Sensitivity Model 2 additionally incorporated these variables as an exploratory treatment-exposure-adjusted analysis to examine the extent to which the estimated strategy-level association was sensitive to differences in actual rehabilitation exposure. Because conditioning on post-initiation variables may introduce overadjustment or mediator-related bias, Sensitivity Model 2 was not interpreted as estimating a controlled direct effect of the rehabilitation strategy or as isolating the independent biological effect of any individual intervention component. An additional exploratory within-group treatment-exposure analysis was conducted among participants in the multimodal rehabilitation group with evaluable follow-up outcomes. The actual number of treatment sessions received and actual treatment duration were examined separately as continuous exposure variables. Follow-up NRS pain score, ODI score, PAC-SYM score, and weekly bowel-movement frequency were modeled as dependent variables. Models were adjusted for age, BMI, postpartum time, mode of delivery, parity, baseline NRS score, baseline ODI score, baseline PAC-SYM score, baseline weekly bowel-movement frequency, baseline Oxford pelvic floor muscle strength grade, analgesic use, laxative use, and the observed baseline-to-follow-up interval. HC3 heteroscedasticity-consistent standard errors were used. Because session number and treatment duration represented correlated measures of rehabilitation exposure, they were entered into separate models rather than simultaneously. Because both exposure measures were determined after treatment initiation, these analyses were considered exploratory and were not interpreted as estimating causal dose-response effects.

To assess the influence of heterogeneous follow-up timing, a restricted-window sensitivity analysis was conducted among participants whose outcome assessment occurred 35–49 days after baseline assessment, representing a study-defined 6-week follow-up window of ±1 week. The same baseline-adjusted outcome models were repeated in this restricted cohort.

To further assess the robustness of the findings to baseline treatment-selection differences, propensity score matching was performed in the 352-participant treatment-initiation cohort. The propensity score was estimated using a multivariable logistic regression model with rehabilitation strategy as the dependent variable and clinically selected pre-treatment covariates defined for the present retrospective analysis as predictors, including age, BMI, postpartum time, mode of delivery, parity, neonatal birth weight, breastfeeding status, episiotomy, degree of perineal laceration, baseline NRS score, baseline ODI score, baseline PAC-SYM score, baseline weekly defecation frequency, Bristol Stool Form Scale score, Oxford pelvic floor muscle strength grade, PFDI-20 score, duration of low back pain, duration of constipation, analgesic use, and laxative use.

Propensity score matching was repeated using the 352-participant treatment-initiation cohort. Propensity scores were estimated using clinically selected pre-treatment covariates defined for the present retrospective analysis. One-to-one matching without replacement was performed using propensity score caliper matching combined with additional restrictions on baseline covariate similarity to improve balance. Matching specifications were evaluated solely according to baseline covariate balance and retention of the matched sample, without reference to outcome data. Covariate balance was assessed using absolute standardized mean differences (SMDs), and an absolute SMD <0.10 was considered to indicate acceptable post-matching balance. Hypothesis-testing *P* values were not used to determine covariate balance. The final matching specification used a caliper of 0.29 standard deviations of the logit of the propensity score, exact matching on mode of delivery, and Mahalanobis-distance matching on age, BMI, baseline PAC-SYM score, and baseline ODI score within the propensity-score caliper. Covariate balance was initially evaluated in the full propensity score-matched treatment-initiation cohort. Because matched outcome analyses were subsequently restricted to participants with evaluable follow-up assessments, all baseline SMDs were recalculated separately in the outcome-available matched subset. If any baseline characteristic had an absolute SMD ≥0.10 after restriction to the outcome-available subset, that variable was additionally included as a covariate in the matched outcome model. In the present analysis, baseline Bristol Stool Form Scale score and baseline Oxford pelvic floor muscle strength grade met this criterion. Continuous matched outcomes were therefore analyzed using regression models additionally adjusted for these two variables, and supportive binary responder outcomes were analyzed using correspondingly adjusted logistic regression models. Robust standard errors accounting for matched-set clustering were used.

In addition, inverse probability of treatment weighting (IPTW) was performed as a sensitivity analysis using the same propensity-score model specified for the PSM analysis. Stabilized treatment weights were calculated as the marginal probability of the treatment actually received divided by the corresponding individual propensity-score probability. To limit the influence of extreme observations, stabilized weights were truncated at the 1st and 99th percentiles. Positivity was evaluated by examining the distribution and overlap of estimated propensity scores between rehabilitation groups. The distribution of stabilized weights was summarized using the minimum, maximum, and selected percentiles. Covariate balance before and after weighting was assessed using absolute standardized mean differences (SMDs), with an absolute SMD <0.10 considered acceptable. The effective weighted sample size was calculated as $\mathrm{ESS}=\frac{{\left(\sum_{i}\;w_{i}\right)}^{2}}{\sum_{i}\;w_{i}^{2}}$.

Propensity scores and treatment weights were estimated in the full treatment-initiation cohort of 352 participants. IPTW outcome analyses were restricted to the 300 participants with evaluable follow-up outcomes; missing follow-up outcomes were not imputed within the IPTW analysis. Potential bias arising from differential follow-up was addressed separately by the IPCW analysis and landmark-anchored MNAR sensitivity analysis described above. Weighted linear and logistic regression models used robust sandwich variance estimates. Exploratory subgroup analyses were conducted for the supportive dual responder outcome, defined as simultaneous achievement of an NRS reduction of ≥2 points and a PAC-SYM reduction of ≥0.6 points. These subgroup analyses were not prospectively specified in a registered protocol or statistical analysis plan. The subgroup variables—postpartum time (≤12 vs. >12 weeks), baseline pain severity (NRS <6 vs. ≥6), baseline constipation symptom severity (PAC-SYM <2.0 vs. ≥2.0), and mode of delivery (cesarean vs. vaginal delivery)—were selected for their clinical relevance within the retrospective analysis. Accordingly, all subgroup findings were considered exploratory and hypothesis-generating. Within each subgroup stratum, the association between rehabilitation strategy and dual response was estimated using logistic regression and reported as an adjusted odds ratio (OR) with a 95% confidence interval (CI). The subgroup models were adjusted for age and BMI. Treatment-by-subgroup interaction terms were used to formally test for effect modification. Because these analyses were exploratory and the study was not specifically powered to detect treatment-by-subgroup interactions, nonsignificant interaction *P values* were interpreted as absence of statistically detectable effect modification rather than evidence that treatment effects were equivalent or consistent across subgroups. All tests were two-sided, and *P* < 0.05 was considered statistically significant.

## 3 Results

### 3.1 Participant flow and baseline characteristics

A total of 410 postpartum women were screened during the study period. Fifty-eight were excluded before treatment initiation because they did not meet the study-defined baseline eligibility criteria, leaving 352 women who initiated rehabilitation and constituted the treatment-initiation cohort. Of these, 170 initiated conventional active rehabilitation comparator and 182 initiated multimodal pelvic floor rehabilitation.

Among the 170 women who initiated conventional active rehabilitation comparator, 158 (92.9%) completed the planned rehabilitation course and 12 (7.1%) discontinued rehabilitation before planned completion. Among the 182 women who initiated multimodal rehabilitation, 172 (94.5%) completed the planned course and 10 (5.5%) discontinued before planned completion. Evaluable follow-up assessments were available for 144 women (84.7%) in the conventional active rehabilitation comparator group and 156 women (85.7%) in the multimodal rehabilitation group, whereas 26 women in each group had no evaluable follow-up outcome assessment. Participant flow is shown in Figure 1, and detailed reasons for treatment discontinuation and unavailable follow-up are presented in Supplementary Table S1.

**Figure 1:**
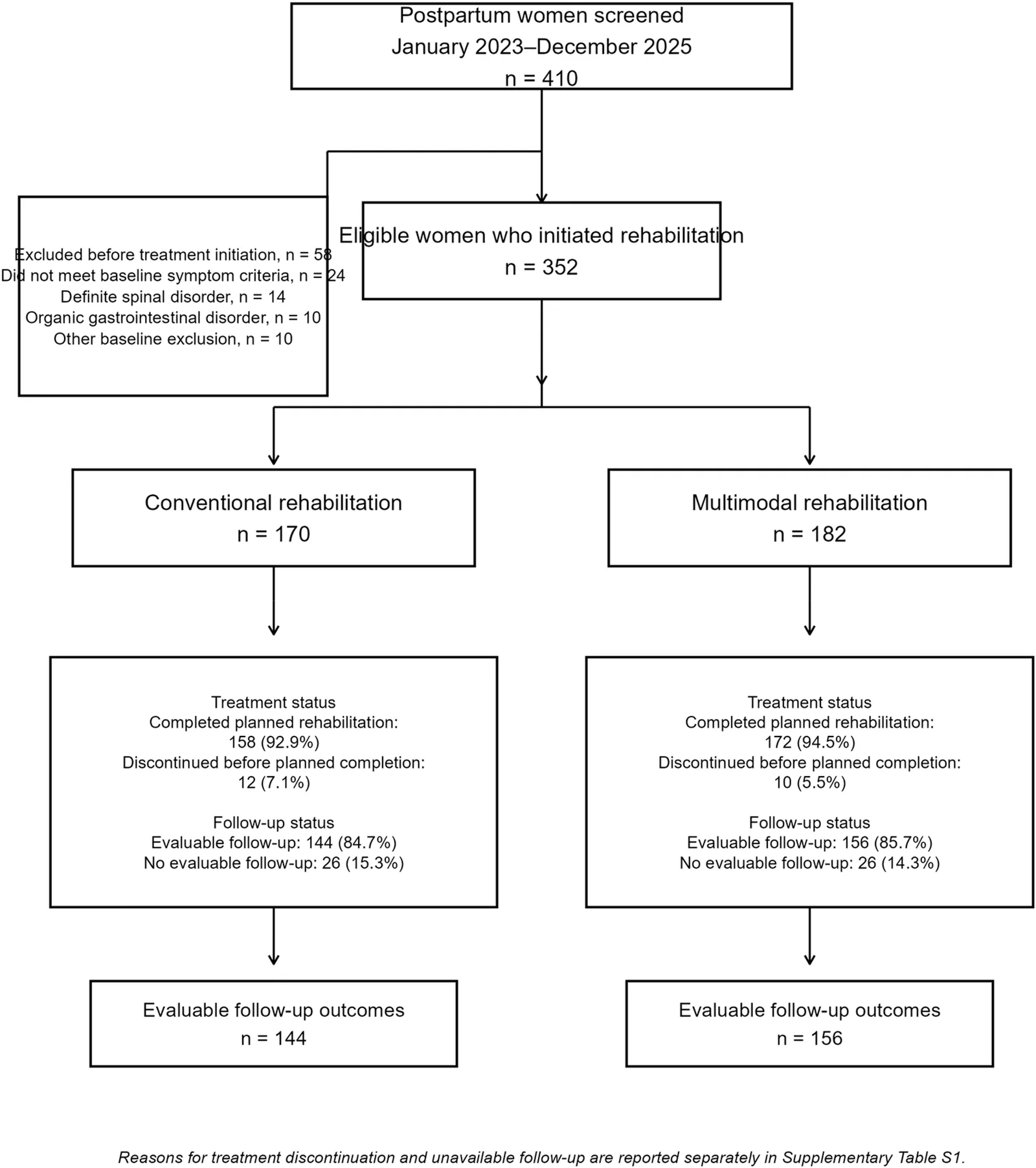
Flow diagram of cohort construction, treatment completion, and follow-up availability.

Baseline characteristics of the treatment-initiation cohort are presented in Table 1. Among the 352 women who initiated rehabilitation, 170 initiated conventional active rehabilitation comparator and 182 initiated multimodal pelvic floor rehabilitation. Although several baseline characteristics were similar between the groups, meaningful imbalance based on standardized mean differences (SMD >0.10) remained for mode of delivery, parity, neonatal birth weight, episiotomy, degree of perineal laceration, baseline PAC-SYM score, baseline Oxford pelvic floor muscle strength grade, baseline PFDI-20 score, and analgesic use. The largest imbalance was observed for baseline PAC-SYM score (SMD = 0.489), which was higher in the multimodal rehabilitation group than in the conventional active rehabilitation comparator group (1.99 ± 0.43 vs. 1.78 ± 0.41, *P* < 0.001). The multimodal group also had lower baseline Oxford pelvic floor muscle strength (2.50 ± 0.76 vs. 2.68 ± 0.85, *P* = 0.037) and higher PFDI-20 scores (55.57 ± 9.12 vs. 53.17 ± 9.51, *P* = 0.016). These baseline imbalances supported the use of multivariable adjustment and propensity score-based sensitivity analyses.

**Table 1:** Baseline characteristics of the treatment-initiation cohort variable.

Variable	Overall (*n* = 352)	Conventional active rehabilitation comparator (*n* = 170)	Multimodal rehabilitation (*n* = 182)	*P value*	SMD
Age, years	31.14 ± 4.42	30.97 ± 4.50	31.31 ± 4.35	0.472	0.076
BMI, kg/m^2^	23.72 ± 2.68	23.76 ± 2.72	23.68 ± 2.64	0.78	0.031
Postpartum time, weeks	14.46 ± 5.87	14.41 ± 5.87	14.52 ± 5.89	0.861	0.019
Mode of delivery				0.114	0.181
Cesarean section	113 (32.1)	62 (36.5)	51 (28.0)		
Vaginal delivery	239 (67.9)	108 (63.5)	131 (72.0)		
Parity				0.600	0.108
1	183 (52.0)	93 (54.7)	90 (49.5)		
2	133 (37.8)	60 (35.3)	73 (40.1)		
≥3	36 (10.2)	17 (10.0)	19 (10.4)		
Neonatal birth weight, g	3,309.01 ± 410.25	3,266.65 ± 427.50	3,348.57 ± 390.49	0.062	0.200
Breastfeeding				0.551	0.077
No	91 (25.9)	41 (24.1)	50 (27.5)		
Yes	261 (74.1)	129 (75.9)	132 (72.5)		
Episiotomy				0.033	0.243
No	267 (75.9)	138 (81.2)	129 (70.9)		
Yes	85 (24.1)	32 (18.8)	53 (29.1)		
Degree of perineal laceration				0.746	0.119
Grade 0	215 (61.1)	104 (61.2)	111 (61.0)		
Grade 1	95 (27.0)	46 (27.1)	49 (26.9)		
Grade 2	35 (9.9)	18 (10.6)	17 (9.3)		
Grade 3	7 (2.0)	2 (1.2)	5 (2.7)		
Baseline NRS pain score	5.22 ± 1.25	5.16 ± 1.21	5.27 ± 1.30	0.412	0.088
Baseline ODI score	34.76 ± 8.50	34.33 ± 8.28	35.17 ± 8.70	0.354	0.098
Baseline PAC-SYM score	1.89 ± 0.43	1.78 ± 0.41	1.99 ± 0.43	<0.001	0.489
Baseline weekly defecation frequency	2.11 ± 0.83	2.14 ± 0.83	2.08 ± 0.84	0.501	0.080
Baseline Bristol Stool Form Scale score	2.56 ± 0.86	2.60 ± 0.88	2.52 ± 0.83	0.382	0.098
Baseline Oxford pelvic floor muscle strength grade	2.59 ± 0.81	2.68 ± 0.85	2.50 ± 0.76	0.037	0.226
Baseline PFDI-20 score	54.41 ± 9.37	53.17 ± 9.51	55.57 ± 9.12	0.016	0.258
Duration of low back pain, weeks	9.70 ± 5.21	9.44 ± 5.24	9.95 ± 5.18	0.359	0.097
Duration of constipation, weeks	8.30 ± 4.44	8.28 ± 4.47	8.32 ± 4.42	0.933	0.009
Analgesic use				0.377	0.107
No	247 (70.2)	115 (67.6)	132 (72.5)		
Yes	105 (29.8)	55 (32.4)	50 (27.5)		
Laxative use				0.699	0.055
No	267 (75.9)	131 (77.1)	136 (74.7)		
Yes	85 (24.1)	39 (22.9)	46 (25.3)		

Continuous variables are presented as mean ± standard deviation, and categorical variables are presented as *n* (%). *P values* were obtained using independent-samples t tests for continuous variables and *χ*^2^ or Fisher's exact tests, as appropriate, for categorical variables. Standardized mean differences (SMDs) were used to assess between-group balance, with an absolute SMD >0.10 indicating potentially meaningful imbalance. NRS, Numerical Rating Scale; ODI, Oswestry Disability Index; PAC-SYM, Patient Assessment of Constipation Symptoms; PFDI-20, Pelvic Floor Distress Inventory-20; BMI, body mass index.

To examine potential calendar-time effects, rehabilitation strategy was additionally summarized according to treatment-initiation year. In 2023, 60 women initiated conventional active rehabilitation comparator and 67 initiated multimodal rehabilitation; in 2024, the corresponding numbers were 54 and 57; and in 2025, they were 56 and 58, respectively. The relative distribution of rehabilitation strategies remained similar across the three calendar years (*χ*^2^ = 0.093, *P* = 0.955), with no evidence of a systematic calendar-time shift in treatment utilization (Supplementary Table S3A).

Baseline comparison according to follow-up availability showed a similar distribution of rehabilitation strategy between participants with and without evaluable follow-up (SMD = 0.040). However, modest differences were observed for several baseline characteristics, including age, BMI, postpartum time, and baseline NRS pain score, indicating the possibility of differential follow-up and motivating the inverse-probability-of-follow-up-weighted sensitivity analysis (Supplementary Table S2).

### 3.2 Treatment exposure, adherence, and follow-up timing

Treatment timing, exposure, adherence, and follow-up characteristics are summarized in Supplementary Table S3B. The median interval from baseline assessment to treatment initiation was similar between the conventional and multimodal rehabilitation groups (1.0 [IQR, 0.2–2.0] vs. 1.0 [IQR, 0.0–2.0] days, *P* = 0.746). Actual treatment duration was longer in the multimodal rehabilitation group than in the conventional active rehabilitation comparator group (5.73 ± 1.13 vs. 5.19 ± 1.29 weeks, *P* < 0.001), and participants in the multimodal group received more treatment sessions (median 12.0 [IQR, 9.0–14.0] vs. 5.0 [IQR, 4.0–6.0], *P* < 0.001).

Treatment adherence was similar between the two groups (90.8 ± 9.1% in the multimodal rehabilitation group vs. 89.6 ± 9.8% in the conventional active rehabilitation comparator group, *P* = 0.236). Good adherence, defined as completion of at least 80% of prescribed sessions, was observed in 158 women (86.8%) and 143 women (84.1%), respectively (*P* = 0.473). Planned rehabilitation was completed by 172 women (94.5%) in the multimodal rehabilitation group and 158 women (92.9%) in the conventional active rehabilitation comparator group (*P* = 0.545).

Among participants with evaluable follow-up assessments, the actual baseline-to-follow-up interval was similar between the multimodal and conventional active rehabilitation comparator groups (57.46 ± 14.03 vs. 57.78 ± 13.22 days, *P* = 0.841). Nevertheless, because individual follow-up timing varied, the actual baseline-to-follow-up interval was retained as a covariate in all primary outcome models.

Documented treatment-related adverse events identified through retrospective record review were generally mild and transient. Transient pelvic or perineal discomfort was documented in 2 participants (1.2%) in the conventional active rehabilitation comparator group and 3 participants (1.6%) in the multimodal rehabilitation group, while transient muscle soreness was documented in 3 participants (1.8%) and 4 participants (2.2%), respectively. Mild vaginal irritation was documented in 2 participants (1.1%) receiving multimodal rehabilitation and in none of the participants receiving conventional active rehabilitation. One participant in each group discontinued rehabilitation because of documented treatment intolerance or an adverse event. No serious treatment-related adverse event was documented in the available rehabilitation or follow-up records (Supplementary Table S5).

### 3.3 Primary analysis among participants with evaluable follow-up

Primary baseline-adjusted analyses were performed among the 300 participants with an evaluable post-initiation outcome assessment (Table 2). For the two co-primary outcomes, NRS and ODI, multimodal pelvic floor rehabilitation was associated with more favorable follow-up scores after adjustment for the corresponding baseline outcome, the observed baseline-to-follow-up interval, and clinically selected baseline covariates.

**Table 2:** Primary baseline-adjusted analysis Among participants With evaluable follow-up.

Outcome	Adjusted *β* (95% CI)	*P value*
Follow-up NRS	−1.58 (−1.79 to −1.37)	<0.001
Follow-up ODI	−9.71 (−10.65 to −8.78)	<0.001
Follow-up PAC-SYM	−0.62 (−0.69 to −0.54)	<0.001
Weekly defecation frequency	1.54 (1.38 to 1.69)	<0.001

Analyses included the 300 participants with an evaluable post-initiation follow-up assessment. Models were adjusted for the corresponding baseline outcome, the observed baseline-to-follow-up interval, and clinically selected baseline covariates defined for the present analysis. HC3 heteroscedasticity-consistent standard errors were used. Negative coefficients for NRS, ODI, and PAC-SYM and a positive coefficient for weekly bowel frequency indicate more favorable outcomes associated with multimodal rehabilitation.

Compared with conventional active rehabilitation comparator, multimodal rehabilitation was associated with a 1.58-point lower follow-up NRS score (adjusted *β* = −1.58, 95% CI: −1.79 to −1.37, *P* < 0.001), a 9.71-point lower follow-up ODI score (*β* = −9.71, 95% CI: −10.65 to −8.78, *P* < 0.001), and a 0.62-point lower follow-up PAC-SYM score (*β* = −0.62, 95% CI: −0.69 to −0.54, *P* < 0.001). Multimodal rehabilitation was also associated with 1.54 additional bowel movements per week at follow-up (*β* = 1.54, 95% CI: 1.38–1.69, *P* < 0.001).

### 3.4 Sensitivity analyses for differential follow-up and missing-data assumptions

Stabilized inverse-probability-of-follow-up weighting was used to assess the potential influence of differential follow-up availability. After truncation at the 1st and 99th percentiles, stabilized weights ranged from 0.884 to 1.328, with a mean of 0.998. The effective sample size was 297.6 of the 300 outcome-evaluable participants, indicating minimal loss of information from weighting. The maximum absolute standardized difference between the outcome-evaluable sample and the full treatment-initiation cohort decreased from 0.0327 before weighting to 0.0039 after weighting, and no covariate had an absolute SMD >0.10 after weighting (Supplementary Table S7 and Supplementary Figures S1, S2).

IPCW-weighted estimates were essentially unchanged from the primary analysis. Multimodal rehabilitation was associated with lower follow-up NRS (*β* = −1.59, 95% CI: −1.78 to −1.39, *P* < 0.001), lower ODI (*β* = −9.63, 95% CI: −10.52 to −8.73, *P* < 0.001), lower PAC-SYM (*β* = −0.62, 95% CI: −0.69 to −0.55, *P* < 0.001), and higher weekly bowel-movement frequency (*β* = 1.55, 95% CI: 1.41–1.70, *P* < 0.001).

A *post hoc* landmark-anchored delta sensitivity analysis was additionally performed to assess departures from MAR. Under the 56-day MAR landmark scenario, the estimated associations remained similar to those of the primary analysis. Progressively unfavorable shifts were then imposed on the imputed outcomes of multimodal participants without follow-up. Even under the severe MNAR scenario, multimodal rehabilitation remained associated with lower NRS (*β* = −1.26, 95% CI: −1.50 to −1.02), lower ODI (*β* = −8.17, 95% CI: −9.32 to −7.02), lower PAC-SYM (*β* = −0.51, 95% CI: −0.60 to −0.43), and higher weekly bowel frequency (*β* = 1.25, 95% CI: 1.07–1.43); all *P* < 0.001. Thus, the direction and statistical interpretation of the observed associations were robust to the examined departures from MAR (Supplementary Table S8 and Supplementary Figure S3).

### 3.5 Additional sensitivity analyses for outcome distribution, treatment exposure, and follow-up timing

Distribution-appropriate sensitivity analyses yielded results consistent with the primary continuous-outcome models. For weekly bowel-movement frequency, the Pearson dispersion statistic from the Poisson model was 0.135, providing no evidence of overdispersion. Multivariable Poisson regression with robust standard errors showed that multimodal rehabilitation was associated with a higher follow-up bowel-movement frequency (adjusted IRR = 1.59, 95% CI: 1.51–1.67, *P* < 0.001).

For the bounded PAC-SYM outcome, beta regression after rescaling the score to the 0–1 interval similarly supported the primary finding. The standardized average marginal difference, transformed back to the original PAC-SYM scale, was −0.66 points (95% CI: −0.74 to −0.59, *P* < 0.001) for multimodal vs. conventional active rehabilitation comparator. This estimate was closely consistent with the −0.62-point association obtained from the primary linear model (Supplementary Table S11). In the exploratory treatment-exposure-adjusted sensitivity analysis, further conditioning on actual treatment duration, number of treatment sessions, and treatment adherence attenuated the magnitude of some associations (Table 3). Multimodal rehabilitation remained associated with a lower follow-up NRS score (*β* = −0.92, 95% CI: −1.32 to −0.51, *P* < 0.001), lower follow-up ODI score (*β* = −9.45, 95% CI: −11.29 to −7.61, *P* < 0.001), lower follow-up PAC-SYM score (*β* = −0.39, 95% CI: −0.54 to −0.25, *P* < 0.001), and higher weekly defecation frequency (*β* = 1.02, 95% CI: 0.73–1.31, *P* < 0.001). Because treatment duration, session number, and adherence are post-initiation variables and potential mediators, these estimates should not be interpreted as controlled direct effects. Rather, the attenuation, particularly for NRS and PAC-SYM, indicates that the estimated strategy-level associations were sensitive in part to differences in treatment exposure.

**Table 3:** Sensitivity analyses for treatment exposure and follow-up timing.

Outcome	Exposure-adjusted model *β* (95% CI)	*P value*	Restricted-window model *β* (95% CI)	*P value*
Follow-up NRS	−0.92 (−1.32 to −0.51)	<0.001	−1.80 (−2.20 to −1.40)	<0.001
Follow-up ODI	−9.45 (−11.29 to −7.61)	<0.001	−8.94 (−10.68 to −7.19)	<0.001
Follow-up PAC-SYM	−0.39 (−0.54 to −0.25)	<0.001	−0.59 (−0.74 to −0.43)	<0.001
Weekly defecation frequency	1.02 (0.73 to 1.31)	<0.001	1.51 (1.21 to 1.81)	<0.001

The treatment-exposure-adjusted model was an exploratory sensitivity analysis that additionally conditioned on actual treatment duration, number of treatment sessions, and treatment adherence. Because these variables were measured after treatment initiation and may represent mediators of the rehabilitation-strategy association, this model was not interpreted as estimating a controlled direct effect or the independent effect of individual rehabilitation components.

An additional exploratory treatment-exposure analysis was conducted among the 156 participants in the multimodal rehabilitation group with evaluable follow-up outcomes. In this subgroup, the median number of treatment sessions received was 12 (IQR, 10–14), and the mean actual treatment duration was 5.89 ± 1.00 weeks. Session number and treatment duration were moderately correlated (Pearson *r* = 0.688) and were therefore examined in separate multivariable models.

After multivariable adjustment, each additional treatment session was associated with a lower follow-up NRS score (*β* = −0.083, 95% CI: −0.133 to −0.034, *P* = 0.001), a lower PAC-SYM score (*β* = −0.025, 95% CI: −0.043 to −0.006, *P* = 0.009), and a higher weekly bowel-movement frequency (*β* = 0.056, 95% CI: 0.019 to 0.093, *P* = 0.003). No clear association was observed with follow-up ODI (*β* = −0.086, 95% CI: −0.334 to 0.162, *P* = 0.496). Similarly, each additional week of actual treatment duration was associated with a lower follow-up NRS score (*β* = −0.307, 95% CI: −0.459 to −0.155, *P* < 0.001), a lower PAC-SYM score (*β* = −0.073, 95% CI: −0.138 to −0.008, *P* = 0.029), and a higher weekly bowel-movement frequency (*β* = 0.140, 95% CI: 0.015 to 0.265, *P* = 0.028), whereas the association with ODI was not statistically evident (*β* = −0.483, 95% CI: −1.219 to 0.253, *P* = 0.198). Thus, exploratory exposure-outcome associations were observed for some, but not all, clinical outcomes (Supplementary Table S12).

A restricted-window sensitivity analysis was additionally performed among the 86 participants whose outcome assessments occurred 35–49 days after baseline assessment, including 39 women in the conventional active rehabilitation comparator group and 47 in the multimodal rehabilitation group. Within this restricted follow-up window, multimodal rehabilitation remained associated with a lower follow-up NRS score (*β* = −1.80, 95% CI: −2.20 to −1.40, *P* < 0.001), lower follow-up ODI score (*β* = −8.94, 95% CI: −10.68 to −7.19, *P* < 0.001), lower follow-up PAC-SYM score (*β* = −0.59, 95% CI: −0.74 to −0.43, *P* < 0.001), and higher weekly defecation frequency (*β* = 1.51, 95% CI: 1.21–1.81, *P* < 0.001). These findings were directionally consistent with the primary analysis and suggested that the observed associations were not attributable solely to variation in follow-up timing.

### 3.6 Change-score sensitivity analysis Among participants with evaluable follow-up

Among the 300 participants with evaluable follow-up assessments, change-score sensitivity analyses yielded findings consistent with the primary observed-follow-up analysis. Regarding low back pain-related outcomes, the reduction in NRS pain score was greater in the multimodal pelvic floor rehabilitation group than in the conventional active rehabilitation comparator group (2.58 ± 0.89 vs. 1.07 ± 0.81; between-group difference = 1.51, *P* < 0.001). The reduction in ODI score was also significantly greater in the multimodal pelvic floor rehabilitation group than in the conventional active rehabilitation comparator group (15.46 ± 4.00 vs. 5.76 ± 3.71; between-group difference = 9.70, *P* < 0.001), suggesting that multimodal pelvic floor rehabilitation was associated with greater improvement in postpartum low back pain and related functional limitation.

Regarding constipation-related outcomes, the reduction in PAC-SYM score was significantly greater in the multimodal pelvic floor rehabilitation group than in the conventional active rehabilitation comparator group (1.00 ± 0.33 vs. 0.38 ± 0.28; between-group difference = 0.62, *P* < 0.001). In addition, the increase in weekly defecation frequency was greater in the multimodal pelvic floor rehabilitation group than in the conventional active rehabilitation comparator group (1.99 ± 0.66 vs. 0.45 ± 0.55; between-group difference = 1.54, *P* < 0.001), as shown in Table 4. Group-level mean NRS, ODI, and PAC-SYM scores at baseline and follow-up are displayed in Figure 2 with 95% confidence intervals. The connecting lines are provided only to facilitate visual comparison of the group-level mean changes and do not depict individual participant trajectories.

**Table 4:** Comparison of changes in continuous outcome measures before and after treatment between the two groups.

Outcome	Conventional active rehabilitation before treatment	Conventional active rehabilitation after treatment	Change in the conventional active rehabilitation group	Multimodal group before treatment	Multimodal group after treatment	Change in multimodal group	Between-group difference	*P* value
NRS pain score	5.14 ± 1.16	4.07 ± 1.26	1.07 ± 0.81	5.34 ± 1.31	2.76 ± 1.51	2.58 ± 0.89	1.51	<0.001
ODI score	34.28 ± 8.29	28.52 ± 8.92	5.76 ± 3.71	35.34 ± 8.89	19.88 ± 9.04	15.46 ± 4.00	9.70	<0.001
PAC-SYM constipation symptom score	1.79 ± 0.40	1.41 ± 0.48	0.38 ± 0.28	1.96 ± 0.42	0.96 ± 0.49	1.00 ± 0.33	0.62	<0.001
Weekly defecation frequency	2.17 ± 0.84	2.62 ± 1.02	0.45 ± 0.55	2.10 ± 0.84	4.09 ± 1.11	1.99 ± 0.66	1.54	<0.001

Continuous variables are presented as mean ± standard deviation. Changes were calculated as the pre-treatment/baseline value minus the post-treatment/follow-up value; for weekly defecation frequency, the change was calculated as the post-treatment value minus the pre-treatment value. The between-group difference was calculated as the change in the multimodal pelvic floor rehabilitation group minus the change in the conventional active rehabilitation comparator group. The *P* value represents the comparison of changes between the two groups.

**Figure 2:**
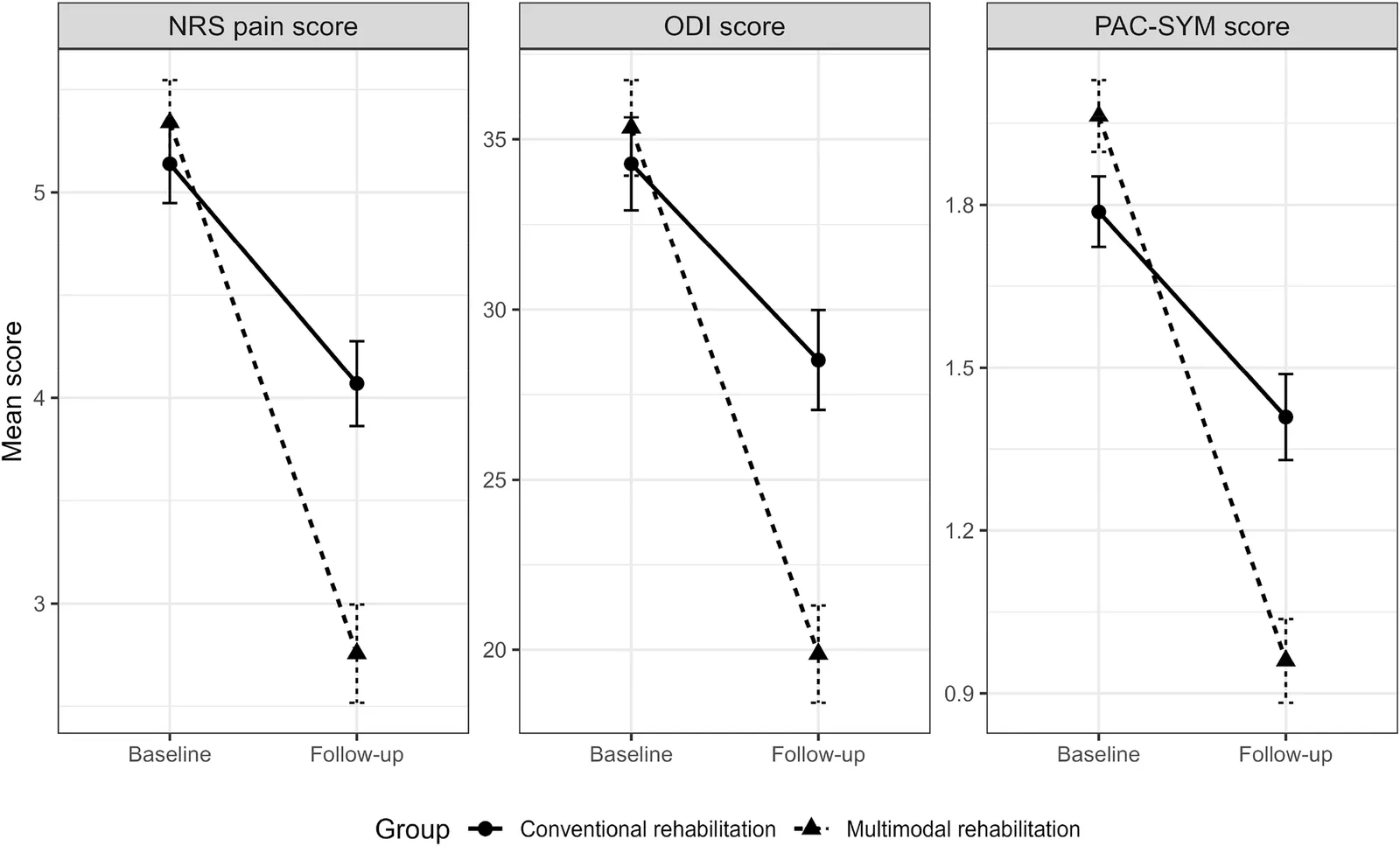
Group-level mean NRS, ODI, and PAC-SYM scores at baseline and follow-up.

Points represent the group means at baseline and follow-up, and error bars represent 95% confidence intervals (CIs) around the group means. Lines connect the two group-level means only to facilitate visual comparison of average changes between baseline and follow-up and do not represent individual participant trajectories. NRS, Numerical Rating Scale; ODI, Oswestry Disability Index; PAC-SYM, Patient Assessment of Constipation Symptoms.

### 3.7 Supportive binary responder analyses

Because the binary responder endpoints were supportive, emphasis was placed on absolute responder rates and between-group risk differences rather than on relative effect estimates. Pain response was observed in 40 of 144 women (27.8%) receiving conventional active rehabilitation comparator and 141 of 156 women (90.4%) receiving multimodal rehabilitation, corresponding to an absolute responder-rate difference of 62.6 percentage points (95% CI: 53.0–70.2).

PAC-SYM MID response was observed in 37 women (25.7%) and 136 women (87.2%), respectively, corresponding to an absolute difference of 61.5 percentage points (95% CI: 51.6–69.3). Bowel-frequency improvement of ≥2 bowel movements per week occurred in 4 women (2.8%) and 121 women (77.6%), respectively, corresponding to an absolute difference of 74.8 percentage points (95% CI: 66.5–80.9). Dual pain plus PAC-SYM MID response occurred in 13 women (9.0%) and 126 women (80.8%), respectively, corresponding to an absolute difference of 71.7 percentage points (95% CI: 62.7–78.3) (Table 5).

**Table 5:** Absolute responder rates and between-group risk differences among participants With evaluable follow-up.

Outcome	Conventional active rehabilitation comparator (*n* = 144)	Multimodal rehabilitation (*n* = 156)	Absolute risk difference, percentage points (95% CI)	*P value*
Pain responder, NRS reduction ≥2 points	40 (27.8)	141 (90.4)	62.6 (53.0 to 70.2)	<0.001
PAC-SYM MID responder, reduction ≥0.6 points	37 (25.7)	136 (87.2)	61.5 (51.6 to 69.3)	<0.001
Bowel-frequency improvement, increase ≥2/week	4 (2.8)	121 (77.6)	74.8 (66.5 to 80.9)	<0.001
Dual pain + PAC-SYM MID responder	13 (9.0)	126 (80.8)	71.7 (62.7 to 78.3)	<0.001

Data are presented as *n* (%). Absolute risk differences were calculated as the responder proportion in the multimodal rehabilitation group minus that in the conventional active rehabilitation comparator group and are expressed in percentage points with 95% confidence intervals. These unadjusted absolute differences are presented to improve clinical interpretability and should not be interpreted as causal treatment effects. Adjusted odds ratios are reported separately as secondary relative measures. NRS, Numerical Rating Scale; PAC-SYM, Patient Assessment of Constipation Symptoms; MID, minimal important difference.

Sensitivity analyses using alternative PAC-SYM response thresholds of an absolute reduction of ≥0.75 points and a relative reduction of ≥30% yielded directionally consistent findings (Supplementary Table S6).

### 3.8 Complete-case change-score regression sensitivity analysis

As an additional complete-case sensitivity analysis, multivariable linear regression models were fitted using change scores as dependent variables. In Sensitivity Model 1, after adjustment for clinically selected pre-treatment baseline covariates defined for the present analysis, multimodal pelvic floor rehabilitation remained associated with greater observed improvements in the continuous outcomes. Compared with the conventional active rehabilitation comparator group, the multimodal rehabilitation group showed an additional 1.58-point improvement in NRS pain score (95% CI: 1.38–1.78, *P* < 0.001), a 9.71-point improvement in ODI score (95% CI: 8.81–10.61, *P* < 0.001), a 0.62-point improvement in PAC-SYM score (95% CI: 0.54–0.69, *P* < 0.001), and an increase of 1.54 bowel movements per week (95% CI: 1.39–1.68, *P* < 0.001).

In Sensitivity Model 2, after further adjustment for actual treatment duration, number of treatment sessions, and treatment adherence, the associations between multimodal pelvic floor rehabilitation and all continuous change-score outcomes remained statistically evident. The multimodal rehabilitation group showed an additional 0.93-point improvement in NRS pain score (95% CI: 0.60–1.27, *P* < 0.001), a 9.39-point improvement in ODI score (95% CI: 7.82–10.95, *P* < 0.001), a 0.40-point improvement in PAC-SYM score (95% CI: 0.28–0.53, *P* < 0.001), and an increase of 1.01 bowel movements per week (95% CI: 0.77–1.26, *P* < 0.001), as shown in Table 6.

**Table 6:** Complete-case change-score regression sensitivity analysis.

Outcome variable	Sensitivity model 1	*P* value	Sensitivity model 2	*P* value
	*β* (95% CI)		*β* (95% CI)	
Improvement in NRS pain score	1.58 (1.38–1.78)	<0.001	0.93 (0.60–1.27)	<0.001
Improvement in ODI score	9.71 (8.81–10.61)	<0.001	9.39 (7.82–10.95)	<0.001
Improvement in PAC-SYM constipation symptom score	0.62 (0.54–0.69)	<0.001	0.40 (0.28–0.53)	<0.001
Increase in weekly defecation frequency	1.54 (1.39–1.68)	<0.001	1.01 (0.77–1.26)	<0.001

The conventional active rehabilitation comparator group was used as the reference group. A *β* coefficient >0 indicates a greater improvement in the multimodal rehabilitation group than in the conventional active rehabilitation comparator group. Sensitivity Model 1 was adjusted for age, BMI, postpartum time, mode of delivery, parity, baseline NRS pain score, baseline ODI score, baseline PAC-SYM score, baseline weekly defecation frequency, baseline Oxford pelvic floor muscle strength grade, analgesic use, and laxative use. Sensitivity Model 2 additionally adjusted for actual treatment duration, number of treatment sessions, and treatment adherence. These complete-case change-score models were secondary sensitivity analyses and were not the primary continuous outcome analyses. NRS, Numerical Rating Scale; ODI, Oswestry Disability Index; PAC-SYM, Patient Assessment of Constipation Symptoms.

### 3.9 Secondary adjusted logistic and penalized sensitivity analyses of responder outcomes

Given the large absolute differences in responder proportions and sparse cells for some endpoints, adjusted odds ratios were interpreted as secondary relative measures rather than as the principal measures of clinical effect. In secondary supportive analyses, multivariable logistic regression showed that multimodal rehabilitation was associated with a higher likelihood of achieving each binary responder outcome. In Sensitivity Model 1, multimodal rehabilitation was associated with pain response (OR = 33.33, 95% CI: 15.70–70.75, *P* < 0.001), PAC-SYM MID response (OR = 21.48, 95% CI: 11.17–41.30, *P* < 0.001), bowel-frequency improvement of ≥2 bowel movements per week (OR = 176.84, 95% CI: 53.72–582.15, *P* < 0.001), and dual pain plus PAC-SYM response (OR = 55.01, 95% CI: 24.73–122.37, *P* < 0.001).

After further adjustment for actual treatment duration, number of treatment sessions, and treatment adherence in Sensitivity Model 2, the associations were attenuated but remained statistically evident for pain response (OR = 12.04, 95% CI: 2.76–52.44, *P* < 0.001), PAC-SYM MID response (OR = 7.49, 95% CI: 1.99–28.23, *P* = 0.003), bowel-frequency improvement (OR = 46.70, 95% CI: 7.99–272.86, *P* < 0.001), and dual response (OR = 16.83, 95% CI: 4.33–65.37, *P* < 0.001) (Table 7).

**Table 7:** Logistic regression analysis of supportive binary responder outcomes.

Outcome	Sensitivity model 1 OR (95% CI)	*P value*	Sensitivity model 2 OR (95% CI)	*P value*
Pain responder	33.33 (15.70–70.75)	<0.001	12.04 (2.76–52.44)	<0.001
PAC-SYM MID responder (≥0.6)	21.48 (11.17–41.30)	<0.001	7.49 (1.99–28.23)	0.003
Bowel-frequency improvement ≥2/week	176.84 (53.72–582.15)	<0.001	46.70 (7.99–272.86)	<0.001
Dual pain + PAC-SYM MID response	55.01 (24.73–122.37)	<0.001	16.83 (4.33–65.37)	<0.001

The conventional active rehabilitation comparator group was used as the reference group. These logistic regression analyses were secondary supportive analyses rather than primary outcome analyses. Sensitivity Model 1 was adjusted for clinically selected pre-treatment baseline covariates. Sensitivity Model 2 additionally adjusted for actual treatment duration, number of treatment sessions, and treatment adherence. PAC-SYM MID response was defined as an absolute reduction of ≥0.6 points. Bowel-frequency improvement was evaluated separately and was defined as an increase of ≥2 bowel movements per week. Dual response required both an NRS reduction of ≥2 points and a PAC-SYM reduction of ≥0.6 points.

Because the conventional logistic estimates were large and bowel-frequency improvement included only four responders in the conventional group, Firth penalized logistic regression was performed as a sparse-data sensitivity analysis. The penalized estimates were attenuated but remained large and directionally consistent: OR = 26.06 (95% CI: 13.27–55.53) for pain response, OR = 17.53 (95% CI: 9.64–33.50) for PAC-SYM MID response, OR = 105.08 (95% CI: 40.24–353.42) for bowel-frequency improvement, and OR = 39.80 (95% CI: 19.75–87.81) for dual pain plus PAC-SYM MID response; all *P* < 0.001 (Supplementary Table S11). Given the magnitude of these relative estimates, clinical interpretation was based primarily on the absolute responder rates and risk differences presented in Table 5.

### 3.10 Covariate balance and outcome analysis after propensity score matching

Propensity score matching was repeated using the reconstructed treatment-initiation cohort. After 1:1 matching, 238 participants were retained, including 119 women in the conventional active rehabilitation comparator group and 119 women in the multimodal rehabilitation group.

Covariate balance in the full matched treatment-initiation cohort was evaluated using absolute standardized mean differences rather than hypothesis-testing *P* values. The revised matching procedure substantially improved measured baseline balance between the two groups. Among the 238 matched treatment initiators, no measured baseline covariate exceeded the absolute SMD threshold of 0.10. The maximum post-matching absolute SMD was 0.095 for BMI, followed by 0.084 for baseline ODI and 0.076 for neonatal birth weight. Thus, all measured baseline covariates met the balance criterion in the full matched treatment-initiation cohort (Table 8 and Figure 3). Selected baseline characteristics and their post-matching SMDs are presented in Table 8, whereas the balance assessment for all covariates included in the propensity-score model is presented in Figure 3.

**Table 8:** Baseline characteristics of the full propensity score-matched treatment-initiation cohort.

Variable	Conventional active rehabilitation comparator (*n* = 119)	Multimodal rehabilitation (*n* = 119)	SMD
Age, years	31.29 ± 4.63	31.29 ± 4.30	0.000
BMI, kg/m^2^	23.89 ± 2.64	23.64 ± 2.82	0.095
Postpartum time, weeks	14.54 ± 5.89	14.56 ± 6.00	0.004
Mode of delivery			0.000
Cesarean section	39 (32.8)	39 (32.8)	
Vaginal delivery	80 (67.2)	80 (67.2)	
Parity			0.028
1	65 (54.6)	64 (53.8)	
2	42 (35.3)	42 (35.3)	
≥3	12 (10.1)	13 (10.9)	
Neonatal birth weight, g	3,324.29 ± 428.26	3,293.19 ± 384.28	0.076
Baseline NRS	5.15 ± 1.10	5.20 ± 1.31	0.040
Baseline ODI	34.39 ± 7.96	35.10 ± 8.90	0.084
Baseline PAC-SYM	1.86 ± 0.41	1.87 ± 0.44	0.031
Baseline Oxford strength	2.66 ± 0.80	2.66 ± 0.72	0.011
Baseline PFDI-20	54.43 ± 9.60	54.33 ± 8.99	0.010
Duration of low back pain, weeks	9.66 ± 5.20	9.57 ± 5.12	0.016

This table describes the full propensity score-matched treatment-initiation cohort before restriction according to follow-up availability. Continuous variables are presented as mean ± standard deviation and categorical variables as *n* (%). Covariate balance after propensity score matching was evaluated using absolute standardized mean differences (SMDs). An absolute SMD <0.10 was considered to indicate acceptable balance. Hypothesis-testing *P values* were not used to determine post-matching balance. NRS, Numerical Rating Scale; ODI, Oswestry Disability Index; PAC-SYM, Patient Assessment of Constipation Symptoms; PFDI-20, Pelvic Floor Distress Inventory-20; BMI, body mass index.

**Figure 3:**
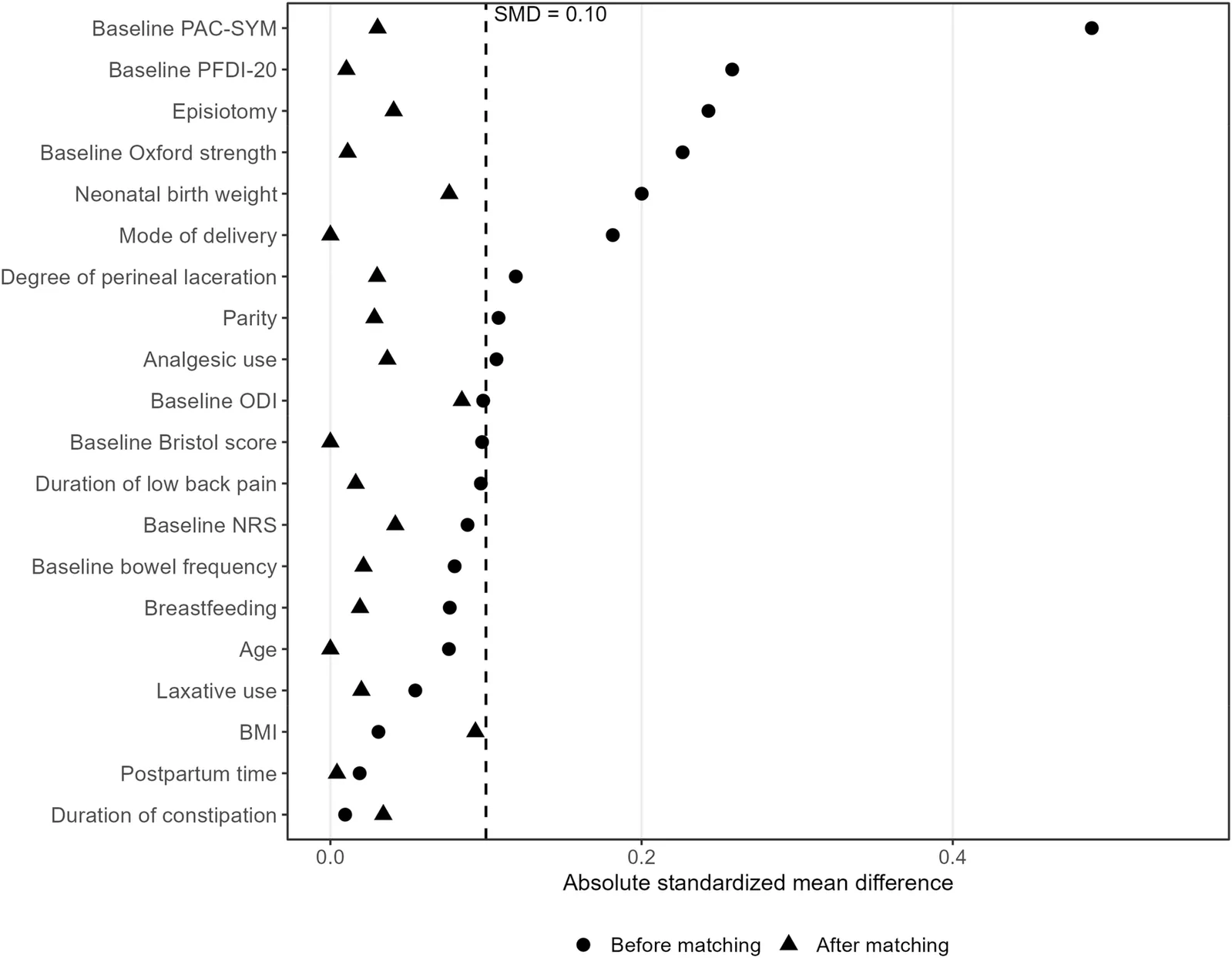
Covariate balance before and after propensity score matching.

Among the 238 matched treatment initiators, evaluable follow-up outcome data were available for 102 women in the conventional active rehabilitation comparator group and 104 women in the multimodal rehabilitation group. Because exclusion of matched participants without evaluable follow-up could potentially alter the original covariate balance, baseline SMDs were recalculated in this 206-participant outcome-available matched subset. Most measured baseline characteristics remained well balanced; however, modest residual imbalance was observed for baseline Bristol Stool Form Scale score (SMD = 0.134) and baseline Oxford pelvic floor muscle strength grade (SMD = 0.125). The maximum absolute SMD was 0.134 (Supplementary Table S9).

Accordingly, matched outcome models were additionally adjusted for baseline Bristol Stool Form Scale score and baseline Oxford pelvic floor muscle strength grade. After this additional adjustment, multimodal rehabilitation remained associated with greater improvement in NRS (adjusted *β* = 1.59, 95% CI: 1.35–1.83, *P* < 0.001), ODI (*β* = 9.47, 95% CI: 8.52–10.42, *P* < 0.001), PAC-SYM (*β* = 0.61, 95% CI: 0.53–0.70, *P* < 0.001), and weekly bowel-movement frequency (*β* = 1.57, 95% CI: 1.40–1.75, *P* < 0.001). Supportive adjusted responder analyses yielded the same overall interpretation (Table 9).

**Table 9:** Outcome analysis among propensity score-matched participants With evaluable follow-up.

Outcome	Conventional active rehabilitation comparator (*n* = 102)	Multimodal rehabilitation (*n* = 104)	Adjusted effect estimate (95% CI)	*P value*
Improvement in NRS	1.10 ± 0.81	2.67 ± 0.93	*β* = 1.59 (1.35–1.83)	<0.001
Improvement in ODI	5.91 ± 3.84	15.44 ± 3.80	*β* = 9.47 (8.52–10.42)	<0.001
Improvement in PAC-SYM	0.37 ± 0.29	0.98 ± 0.33	*β* = 0.61 (0.53–0.70)	<0.001
Increase in weekly defecation frequency	0.44 ± 0.57	2.01 ± 0.66	*β* = 1.57 (1.40–1.75)	<0.001
Pain responder, NRS reduction ≥2 points	29 (28.4)	95 (91.3)	OR = 26.60 (11.86–59.63)	<0.001
PAC-SYM MID responder, reduction ≥0.6 points	25 (24.5)	90 (86.5)	OR = 21.17 (9.74–46.05)	<0.001
Bowel-frequency improvement, increase ≥2/week	4 (3.9)	82 (78.8)	OR = 94.59 (30.90–289.57)	<0.001
Dual pain + PAC-SYM MID responder	10 (9.8)	85 (81.7)	OR = 50.79 (21.05–122.55)	<0.001

The full propensity score-matched treatment-initiation cohort consisted of 238 participants, with 119 participants in each rehabilitation group. Because evaluable follow-up outcomes were unavailable for 17 matched participants in the conventional active rehabilitation group and 15 matched participants in the multimodal rehabilitation group, the outcome analyses presented in this table included 102 and 104 participants, respectively. Thus, the smaller denominators reflect post-matching follow-up attrition rather than additional exclusions arising from the matching procedure. After restriction according to follow-up availability, baseline covariate balance was reassessed, and residual imbalance was observed for baseline Bristol Stool Form Scale score (SMD = 0.134) and baseline Oxford pelvic floor muscle strength grade (SMD = 0.125). Accordingly, continuous and binary matched outcome models were additionally adjusted for these two baseline variables. Adjusted *β* coefficients are reported for continuous outcomes and adjusted odds ratios (ORs) for binary outcomes. Robust standard errors accounting for matched-set clustering were used. Full covariate balance in the outcome-available matched subset is presented in Supplementary Table S9.

Absolute standardized mean differences (SMDs) are shown for measured baseline covariates before and after propensity score matching. Circles represent values before matching and triangles represent values after matching. The dashed vertical reference line represents an absolute SMD of 0.10, which was used as the threshold for acceptable covariate balance. No measured baseline covariate remained inadequately balanced after matching, because all post-matching absolute SMDs were <0.10. The largest residual imbalance was observed for BMI (SMD = 0.095), followed by baseline ODI score (SMD = 0.084) and neonatal birth weight (SMD = 0.076).

### 3.11 IPTW-weighted sensitivity analysis and subgroup interaction analysis

Treatment propensity scores showed substantial overlap between the two rehabilitation groups. Estimated propensity scores ranged from 0.077 to 0.890 in the conventional active rehabilitation comparator group and from 0.095 to 0.903 in the multimodal rehabilitation group, with no evidence of marked positivity violations. Before truncation, stabilized treatment weights ranged from 0.523 to 5.446; the 1st, 5th, 50th, 95th, and 99th percentiles were 0.554, 0.604, 0.859, 1.812, and 3.484, respectively. After truncation at the 1st and 99th percentiles, stabilized weights ranged from 0.554 to 3.484, with a mean of 0.998. The effective weighted sample size in the 352-participant treatment-initiation cohort was 289.6.

IPTW substantially improved measured baseline covariate balance. The maximum absolute SMD decreased from 0.489 before weighting to 0.049 after weighting. After IPTW, all clinically selected baseline covariates had absolute SMDs <0.10. The largest residual imbalances were observed for baseline Oxford pelvic floor muscle strength grade (SMD = 0.049), baseline PAC-SYM score (SMD = 0.048), baseline PFDI-20 score (SMD = 0.044), and age (SMD = 0.038). Propensity-score overlap, weight distribution, and covariate-balance diagnostics are presented in Supplementary Table S10 and Supplementary Figure S4.

IPTW outcome analyses were performed among the 300 participants with evaluable follow-up outcomes, corresponding to an effective treatment-weighted sample size of 248.4. Missing follow-up outcomes were not imputed within the treatment-IPTW analysis; their potential influence was evaluated separately using the IPCW and landmark-anchored MNAR analyses described above.

The IPTW-weighted sensitivity analysis yielded results directionally consistent with the other analyses. Weighted linear regression showed greater observed improvements in NRS pain score (*β* = 1.54, 95% CI: 1.33–1.75), ODI score (*β* = 9.70, 95% CI: 8.76–10.64), PAC-SYM score (*β* = 0.61, 95% CI: 0.54–0.69), and weekly defecation frequency (*β* = 1.57, 95% CI: 1.42–1.72) in the multimodal rehabilitation group; all *P* < 0.001.

In weighted logistic regression, multimodal rehabilitation was associated with pain response (OR = 23.77, 95% CI: 11.02–51.23), PAC-SYM MID response (OR = 18.57, 95% CI: 9.40–36.66), bowel-frequency improvement of ≥2 bowel movements per week (OR = 120.99, 95% CI: 39.80–367.79), and dual pain plus PAC-SYM MID response (OR = 41.94, 95% CI: 19.59–89.81); all *P* < 0.001 (Table 10).

**Table 10:** IPTW-weighted sensitivity analysis.

Outcome variable	Model	Effect estimate (95% CI)	*P value*
Improvement in NRS pain score	Weighted linear regression	1.54 (1.33–1.75)	<0.001
Improvement in ODI score	Weighted linear regression	9.70 (8.76–10.64)	<0.001
Improvement in PAC-SYM score	Weighted linear regression	0.61 (0.54–0.69)	<0.001
Increase in weekly defecation frequency	Weighted linear regression	1.57 (1.42–1.72)	<0.001
Pain responder	Weighted logistic regression	23.77 (11.02–51.23)	<0.001
PAC-SYM MID responder (≥0.6)	Weighted logistic regression	18.57 (9.40–36.66)	<0.001
Bowel-frequency improvement ≥2/week	Weighted logistic regression	120.99 (39.80–367.79)	<0.001
Dual pain + PAC-SYM MID response	Weighted logistic regression	41.94 (19.59–89.81)	<0.001

Propensity scores and stabilized treatment weights were estimated in the full treatment-initiation cohort (*n* = 352). Stabilized weights were truncated at the 1st and 99th percentiles. Outcome models included participants with evaluable follow-up outcomes (*n* = 300) and used robust sandwich variance estimates. Missing follow-up outcomes were not imputed in the IPTW analysis; differential follow-up was addressed separately using IPCW and landmark-anchored MNAR sensitivity analyses. Detailed propensity-score overlap, weight-distribution, effective-sample-size, and covariate-balance diagnostics are provided in Supplementary Table S10 and Supplementary Figure S4.

Exploratory subgroup analyses were repeated using the revised dual responder definition, requiring both an NRS reduction of ≥2 points and a PAC-SYM reduction of ≥0.6 points. Adjusted subgroup-specific ORs and 95% CIs are presented in Table 11. There was no statistically significant evidence of effect modification by postpartum time (*P f*or *interaction* = 0.548), baseline pain severity (*P* = 0.605), baseline constipation symptom severity (*P* = 0.077), or mode of delivery (*P* = 0.208). However, these analyses were exploratory and were not specifically powered to detect interaction effects. Several subgroup estimates had wide confidence intervals, particularly among women with baseline PAC-SYM ≥2.0 and among those with cesarean delivery, indicating substantial statistical imprecision. Therefore, the nonsignificant interaction tests should not be interpreted as demonstrating equivalent or consistent associations across subgroups.

**Table 11:** Exploratory subgroup analysis of dual pain and PAC-SYM MID response.

Subgroup	Level	Total *n*	Conventional events/total	Multimodal events/total	Adjusted OR (95% CI)	*P for interaction*
Postpartum time	≤12 weeks	115	7/61	44/54	60.04 (16.10–223.93)	0.548
	>12 weeks	185	6/83	82/102	52.95 (20.08–139.66)	
Baseline pain severity	NRS <6	183	7/89	76/94	50.49 (19.84–128.48)	0.605
	NRS ≥6	117	6/55	50/62	43.52 (13.68–138.45)	
Baseline constipation severity	PAC-SYM <2.0	185	12/104	65/81	34.87 (14.82–82.02)	0.077
	PAC-SYM ≥2.0	115	1/40	61/75	172.33 (21.62–1,373.44)	
Mode of delivery	Cesarean	92	3/50	36/42	129.02 (24.93–667.66)	0.208
	Vaginal	208	10/94	90/114	37.23 (16.02–86.51)	

Dual response was defined as simultaneous achievement of an NRS reduction of ≥2 points and an absolute PAC-SYM reduction of ≥0.6 points. Within each subgroup stratum, adjusted ORs and 95% CIs were estimated using logistic regression models adjusted for age and BMI. Interaction *P values* were obtained from treatment-by-subgroup interaction terms. These subgroup analyses were exploratory and the study was not specifically powered to detect interaction effects. Accordingly, a nonsignificant interaction *P value* should not be interpreted as evidence of equivalence or consistency of the rehabilitation association across subgroup levels. ORs should be interpreted cautiously because several strata contained small numbers of events and consequently had wide confidence intervals. NRS, Numerical Rating Scale; PAC-SYM, Patient Assessment of Constipation Symptoms; OR, odds ratio; CI, confidence interval.

## 4 Discussion

Based on real-world rehabilitation data from postpartum women with low back pain and constipation-related symptoms, this study evaluated the association between multimodal pelvic floor rehabilitation and subsequent pain, disability, and constipation-related outcomes. Importantly, the exposure contrast should be understood at the rehabilitation-strategy level. The study compared multimodal rehabilitation as delivered in routine clinical practice with conventional active rehabilitation and was not designed to isolate the independent biological contribution of biofeedback, electrical stimulation, core stabilization training, behavioral training, or any other individual component.

For the two co-primary outcomes, multimodal rehabilitation was associated with an adjusted 1.58-point lower follow-up NRS score and a 9.71-point lower ODI score. Key secondary supportive outcomes showed an adjusted 0.62-point lower PAC-SYM score and approximately 1.54 additional bowel movements per week. These effect estimates provide complementary information regarding symptom severity, functional limitation, and bowel function. The PAC-SYM difference was similar in magnitude to the published 0.6-point minimal important difference used in chronic constipation populations; however, that threshold has not been specifically validated in postpartum women and should therefore not be interpreted as establishing clinically important benefit in this population. The adjusted group-level mean differences should not be directly equated with individual responder thresholds; for example, the 1.58-point adjusted NRS contrast does not imply that every participant achieved a ≥2-point pain response. Similarly, the approximately 1.54 additional bowel movements per week is clinically interpretable but does not capture other dimensions of constipation, such as stool consistency, straining, or incomplete evacuation.

Supportive responder analyses showed very large between-group differences in pain response, PAC-SYM MID response, bowel-frequency improvement, and dual response. The corresponding odds ratios were consequently large and should be interpreted with particular caution. One important statistical explanation is the marked separation in responder proportions between the groups. Pain response was observed in 27.8% of participants receiving conventional active rehabilitation compared with 90.4% receiving multimodal rehabilitation, while bowel-frequency improvement occurred in only 2.8% vs. 77.6%, respectively. In particular, only four participants in the conventional group achieved the bowel-frequency responder criterion, resulting in a sparse cell and a very large relative odds estimate with a wide confidence interval. Therefore, the absolute responder rates and between-group risk differences were considered more clinically interpretable than the corresponding odds ratios.

Several features of the study may also have amplified these observed responder contrasts. Outcome assessors were not blinded to rehabilitation strategy, participants were aware of the treatment they received, and in some cases outcome assessments were performed by personnel involved in rehabilitation delivery. Differential patient expectations, performance effects, therapist–patient interaction, recall, and interviewer-related measurement effects therefore cannot be excluded. These concerns are particularly relevant because the responder endpoints were derived predominantly from patient-reported pain, disability, constipation symptoms, and bowel-movement frequency. Participants receiving multimodal rehabilitation also had substantially greater supervised treatment exposure and professional contact than those receiving conventional active rehabilitation. Repeated therapist contact, reinforcement of exercises and behavioral recommendations, monitoring of progress, and greater opportunities to support adherence could themselves contribute to symptom improvement and reporting of improvement.

Consistent with this interpretation, further adjustment for actual treatment duration, number of treatment sessions, and treatment adherence substantially attenuated the responder associations, although they remained statistically evident. Thus, the magnitude of the responder odds ratios should not be interpreted as representing the specific biological effect of the multimodal components themselves. Rather, the observed associations may reflect a combination of the rehabilitation components, greater overall treatment exposure and clinician contact, patient expectations and performance effects, and residual or unmeasured confounding inherent in the nonrandomized observational design. Accordingly, these binary responder analyses were regarded as supportive, and the continuous outcomes, absolute responder rates, and risk differences were given greater weight in clinical interpretation. The exploratory analysis restricted to the multimodal rehabilitation group provided additional information regarding the potential contribution of treatment exposure. A greater number of treatment sessions and longer actual treatment duration were associated with more favorable NRS, PAC-SYM, and bowel-frequency outcomes, whereas no clear exposure-outcome association was observed for ODI. Thus, the pattern was not uniform across all clinical outcomes. Importantly, these findings should not be interpreted as demonstrating a causal dose-response relationship. Session number and treatment duration were post-initiation variables and could themselves have been influenced by adherence, early clinical response, patient motivation, scheduling constraints, clinician decisions, or other unmeasured factors. Reverse causation also cannot be excluded, because early symptom improvement may influence subsequent treatment attendance or duration. Accordingly, this within-group analysis provides exploratory support for treatment intensity as a potential contributor to the observed strategy-level association rather than evidence of an independent biological treatment-dose effect.

The following mechanisms are discussed only as biological plausibility for the multimodal strategy and should not be interpreted as evidence that any individual component independently produced the observed clinical differences. The findings of this study are generally consistent with previous studies. Recent systematic reviews and meta-analyses have shown that pelvic floor muscle training combined with physical therapy may help reduce the severity of low back pain, particularly in women with pregnancy-related or postpartum-related lumbopelvic pain (15). Regarding core stability training, previous studies have suggested that core stability training can improve pelvic floor muscle function, transversus abdominis function, and quality of life in pregnant and postpartum women, which is broadly consistent with the greater ODI improvement observed in the multimodal rehabilitation group; however, the present study cannot attribute this difference specifically to core stability training (16). In addition, studies on postpartum pelvic floor dysfunction have shown that pelvic floor muscle training combined with professional feedback or biofeedback can help improve patients' ability to identify pelvic floor muscle contraction and relaxation and improve some pelvic floor-related symptoms (17). For constipation-related symptoms, postpartum anorectal and pelvic floor disorders are often underestimated, and constipation, difficult defecation, and pelvic floor dysfunction may persist after childbirth (18). Other studies have also shown that delivery-related pelvic floor injury may be associated with the development of constipation (19). Meanwhile, studies on obstructed defecation or defecatory coordination disorders have suggested that biofeedback therapy can improve defecatory coordination and some clinical symptoms (20). Compared with these studies, the present study is characterized by its simultaneous focus on two symptoms that commonly coexist after childbirth, namely low back pain and constipation. By integrating structured pelvic floor muscle training, biofeedback, low-frequency electrical stimulation, core stability training, and defecation behavior guidance into a multimodal rehabilitation program, our findings suggest that receipt of multimodal rehabilitation was associated with greater observed symptom improvement than conventional active rehabilitation comparator comprising routine education, lifestyle guidance, and basic home-based PFMT.

From a mechanistic perspective, postpartum low back pain and constipation may not be completely independent symptoms, but may be jointly influenced by reduced lumbar-pelvic stability, abnormal abdominopelvic pressure regulation, and impaired pelvic floor muscle coordination (21, 22). After pregnancy and childbirth, changes occur in the abdominal wall, pelvic floor muscles, ligaments, fascia, and other supporting structures. Impaired coordinated control among the transversus abdominis, multifidus, diaphragm, and pelvic floor muscles may increase lumbosacral loading, reduce postural control, and contribute to persistent pain (23, 24). Pelvic floor muscle training can enhance active contraction and relaxation of the pelvic floor muscles; biofeedback can help patients identify incorrect exertion patterns; low-frequency electrical stimulation may promote neuromuscular recruitment and muscle strength recovery; and core stability training may improve the control of the transversus abdominis, multifidus, and muscles surrounding the pelvis (25, 26). Meanwhile, constipation-related behavioral interventions, such as diaphragmatic breathing, defecation posture guidance, regular defecation training, dietary fiber intake, and water consumption recommendations, may improve the coordination between abdominal pressure generation and pelvic floor relaxation during defecation, thereby reducing straining and paradoxical anal sphincter contraction (27). These mechanisms provide biological plausibility for the observed associations between multimodal rehabilitation and symptom improvement. However, the present study did not directly measure changes in neuromuscular recruitment, abdominopelvic pressure regulation, pelvic floor coordination, or other potential mediators. Therefore, these mechanisms should be regarded as hypotheses based on previous literature rather than mechanisms demonstrated by the present data.

This study has several limitations. First, this was a single-center retrospective observational study, and rehabilitation strategy was not randomly assigned. Treatment selection reflected shared decision-making between physicians and patients and could be influenced not only by baseline clinical characteristics but also by patient preference, treatment cost and insurance coverage, ability to attend repeated supervised sessions, appointment availability, and access to device-assisted rehabilitation. Although the distribution of rehabilitation strategies remained similar across calendar years and no major changes in the clinical team, equipment, or institutional rehabilitation protocols occurred during 2023–2025, detailed socioeconomic circumstances, motivation, treatment expectations, and other determinants of treatment choice were not systematically recorded. Because multivariable and propensity-score methods can account only for measured covariates, residual selection bias and unmeasured confounding cannot be excluded. Second, the treatment contrast encompassed both differences in rehabilitation content and differences in treatment intensity. Compared with conventional active rehabilitation comparator, the multimodal strategy involved structured therapist-supervised PFMT, biofeedback, electrical stimulation, core stabilization training, constipation-related behavioral training, more frequent professional contact, a greater number of treatment sessions, and a somewhat longer actual treatment duration. Consequently, the observed strategy-level association cannot be attributed specifically to any individual intervention component. The exploratory treatment-exposure-adjusted analysis further conditioned on actual treatment duration, session number, and adherence and attenuated some associations, particularly those for NRS and PAC-SYM. However, these variables occurred after treatment initiation and may themselves be consequences or mediators of the rehabilitation strategy. Conditioning on them may therefore introduce overadjustment or mediator-related bias. Accordingly, this sensitivity analysis should not be interpreted as estimating the direct biological effect of the multimodal components. Rather, the attenuation suggests that differences in treatment exposure may contribute to the overall contrast between rehabilitation strategies. Although the within-multimodal exploratory analysis identified exposure-outcome associations for several outcomes, the absence of a uniform pattern across outcomes and the post-initiation nature of treatment exposure preclude causal interpretation. The independent contributions of individual modalities, professional contact, treatment dose, patient engagement, and treatment expectations cannot be separated using the present observational data. Third, follow-up outcomes were unavailable for 52 of the 352 treatment initiators, and participants with and without evaluable follow-up differed modestly in several baseline characteristics. Although IPCW and landmark-anchored MNAR sensitivity analyses yielded findings broadly consistent with the primary analysis, these approaches rely on assumptions regarding measured determinants of follow-up and cannot eliminate bias arising from unmeasured causes of attrition. Informative loss to follow-up therefore remains possible. Fourth, outcome assessment relied mainly on patient-reported symptom and functional measures. Because assessments were conducted as part of routine clinical care, outcome assessors and participants were aware of the rehabilitation strategy received, and some assessments may have been conducted by rehabilitation personnel involved in treatment delivery. Differential expectations, professional contact, recall, and interviewer-related measurement bias therefore cannot be excluded, particularly given the large between-group differences in the subjective responder outcomes. In addition, the study lacked objective measures of pelvic floor or anorectal function, such as pelvic floor surface electromyography, anorectal manometry, balloon expulsion testing, pelvic floor ultrasonography, assessment of diastasis recti, or objective lumbopelvic muscle-function testing. The study also evaluated primarily short-term outcomes, and the durability of the observed symptom differences remains uncertain. Adverse events were identified retrospectively from routine clinical records rather than through standardized prospective surveillance; therefore, the reported frequencies represent documented events only and should not be interpreted as estimates of the true incidence of treatment-related adverse events. Fifth, participants were characterized as having postpartum constipation-related symptoms rather than Rome IV-defined functional constipation because the required chronicity criterion could not be reliably established retrospectively. In addition, although an absolute PAC-SYM reduction of ≥0.6 points has been reported as a minimal important difference in chronic constipation populations, this threshold has not been specifically validated in postpartum women. Distribution-appropriate and penalized sensitivity analyses did not materially alter the overall interpretation; however, the very large odds ratios for several supportive responder endpoints were accompanied by sparse cells and wide confidence intervals. Absolute responder rates and risk differences were therefore considered more clinically interpretable than the relative odds estimates. These sources of measurement and performance bias may have contributed not only to the direction but also to the unusually large magnitude of the observed responder contrasts, particularly for subjective binary endpoints.

Bowel-frequency improvement was analyzed separately and was not treated as equivalent to PAC-SYM response. Importantly, the observed between-group differences should not be interpreted as causal treatment effects. Residual selection bias and unmeasured confounding may remain despite the multiple adjustment and sensitivity approaches used in this study. Future multicenter prospective studies, ideally with standardized treatment protocols, objective pelvic floor and lumbopelvic assessments, longer follow-up, and evaluation of treatment dose and cost-effectiveness, are needed to clarify the clinical value of multimodal pelvic floor rehabilitation.

In this retrospective observational cohort, multimodal pelvic floor rehabilitation was associated with greater observed improvements in low back pain, functional disability, and constipation-related symptoms compared with the conventional active rehabilitation comparator among postpartum women with concomitant low back pain and constipation. The overall direction of the findings was robust across sensitivity analyses. However, because treatment allocation was nonrandomized and selection bias, residual confounding, and differences in treatment exposure or follow-up cannot be excluded, the findings should not be interpreted as establishing a causal treatment effect. The potential biological mechanisms discussed in this study remain hypotheses and require confirmation in prospective studies with standardized treatment protocols and objective mechanistic assessments.

## Data Availability

The raw data supporting the conclusions of this article will be made available by the authors, without undue reservation.
