# Supplementary material for: Association between multimodal pelvic floor rehabilitation and symptom improvement in postpartum women with low back pain and constipation: a retrospective cohort study
Source: Front Med (Lausanne). 2026 Oct 27;13:1909450. doi: 10.3389/fmed.2026.1909450 (PMC13748217; doi:10.3389/fmed.2026.1909450)
Supplement: Supplementary file 1 [file Table1.docx]

**Supplementary Materials**

**Supplementary Table S1. Treatment Completion and Follow-up Status According to Rehabilitation Strategy**

| **Characteristic** | **conventional active rehabilitation comparator (n=170)** | **Multimodal rehabilitation (n=182)** |
| --- | --- | --- |
| Treatment initiated | 170 (100.0%) | 182 (100.0%) |
| Completed planned rehabilitation | 158 (92.9%) | 172 (94.5%) |
| Discontinued rehabilitation before planned completion | 12 (7.1%) | 10 (5.5%) |
| Evaluable follow-up assessment | 144 (84.7%) | 156 (85.7%) |
| No evaluable follow-up assessment | 26 (15.3%) | 26 (14.3%) |
| Reasons for treatment discontinuation |  |  |
| Patient preference | 4 (33.3%) | 3 (30.0%) |
| Scheduling/work/family constraints | 4 (33.3%) | 3 (30.0%) |
| Symptom improvement before planned completion | 2 (16.7%) | 2 (20.0%) |
| Intolerance/adverse event | 1 (8.3%) | 1 (10.0%) |
| Other/unknown | 1 (8.3%) | 1 (10.0%) |
| Reasons for unavailable follow-up |  |  |
| Unable to contact/lost to follow-up | 11 (42.3%) | 10 (38.5%) |
| Declined follow-up assessment | 6 (23.1%) | 5 (19.2%) |
| Scheduling/family constraints | 5 (19.2%) | 7 (26.9%) |
| Other/unknown | 4 (15.4%) | 4 (15.4%) |

**Note:** Data are presented as n (%) unless otherwise indicated. Percentages for treatment completion and follow-up availability were calculated using the number of participants who initiated the respective rehabilitation strategy as the denominator. Percentages for reasons for treatment discontinuation were calculated among participants who discontinued rehabilitation before planned completion (n=12 in the conventional active rehabilitation comparator group and n=10 in the multimodal rehabilitation group). Percentages for reasons for unavailable follow-up were calculated among participants without an evaluable follow-up assessment (n=26 in each group).

**Supplementary Table S2. Baseline Characteristics According to Follow-up Availability**

| **Variable** | **Follow-up unavailable (n=52)** | **Follow-up available (n=300)** | **SMD** |
| --- | --- | --- | --- |
| Rehabilitation strategy |  |  | 0.04 |
| conventional active rehabilitation comparator | 26 (50.0%) | 144 (48.0%) |  |
| Multimodal rehabilitation | 26 (50.0%) | 156 (52.0%) |  |
| Age, years | 31.81 ± 4.16 | 31.03 ± 4.46 | 0.18 |
| BMI, kg/m² | 24.07 ± 2.54 | 23.66 ± 2.70 | 0.156 |
| Postpartum time, weeks | 15.00 ± 5.98 | 14.37 ± 5.86 | 0.106 |
| Baseline NRS pain score | 5.06 ± 1.33 | 5.24 ± 1.24 | 0.144 |
| Baseline ODI score | 34.37 ± 7.92 | 34.83 ± 8.61 | 0.056 |
| Baseline PAC-SYM score | 1.92 ± 0.52 | 1.88 ± 0.42 | 0.097 |
| Baseline Oxford pelvic floor muscle strength grade | 2.62 ± 0.97 | 2.58 ± 0.78 | 0.036 |

**Note:** Continuous variables are presented as mean ± standard deviation and categorical variables as n (%). Standardized mean differences (SMDs) were used to assess differences in baseline characteristics according to follow-up availability. An absolute SMD >0.10 was considered indicative of potentially meaningful imbalance. NRS, Numerical Rating Scale; ODI, Oswestry Disability Index; PAC-SYM, Patient Assessment of Constipation Symptoms; BMI, body mass index.

**Supplementary Table S3A. Distribution of Rehabilitation Strategy According to Calendar Year**

| **Calendar year** | **conventional active rehabilitation comparator, n (%)** | **Multimodal rehabilitation, n (%)** | **Total** |
| --- | --- | --- | --- |
| 2023 | 60 (47.2) | 67 (52.8) | 127 |
| 2024 | 54 (48.6) | 57 (51.4) | 111 |
| 2025 | 56 (49.1) | 58 (50.9) | 114 |
| Total | 170 (48.3) | 182 (51.7) | 352 |

**Note:** Percentages within each calendar year are presented. The distribution of rehabilitation strategy did not differ significantly across calendar years (Pearson *χ²*=0.093, df=2, *P*=0.955).

**Supplementary Table S3B. Treatment Timing, Exposure, Adherence, and Follow-up Characteristics According to Rehabilitation Strategy**

| **Characteristic** | **conventional active rehabilitation comparator (n=170)** | **Multimodal rehabilitation (n=182)** | ***P value*** |
| --- | --- | --- | --- |
| Baseline assessment date, range | 2023-01-06 to 2025-09-25 | 2023-01-07 to 2025-09-08 | — |
| Treatment initiation date, range | 2023-01-06 to 2025-09-25 | 2023-01-07 to 2025-09-08 | — |
| Baseline-to-treatment initiation interval, days | 1.0 (0.2–2.0) | 1.0 (0.0–2.0) | 0.746 |
| Final treatment session date, range | 2023-02-16 to 2025-11-04 | 2023-02-20 to 2025-10-17 | — |
| Actual treatment duration, weeks | 5.19 ± 1.29 | 5.73 ± 1.13 | <0.001 |
| Number of treatment sessions received | 5.0 (4.0–6.0) | 12.0 (9.0–14.0) | <0.001 |
| Treatment adherence, % | 89.6 ± 9.8 | 90.8 ± 9.1 | 0.236 |
| Good adherence (≥80%), n (%) | 143 (84.1) | 158 (86.8) | 0.473 |
| Completed planned rehabilitation, n (%) | 158 (92.9) | 172 (94.5) | 0.545 |
| Discontinued before planned completion, n (%) | 12 (7.1) | 10 (5.5) | 0.545 |
| Outcome assessment date, range* | 2023-02-25 to 2025-11-18 | 2023-02-21 to 2025-11-01 | — |
| Baseline-to-follow-up interval, days* | 57.78 ± 13.22 | 57.46 ± 14.03 | 0.841 |
| Treatment initiation-to-follow-up interval, days* | 56.37 ± 13.36 | 56.02 ± 14.06 | — |
| Follow-up within 35–49 days, n/N (%)* | 39/144 (27.1%) | 47/156 (30.1%) | — |

**Note:** Data are presented as mean ± standard deviation, median (interquartile range), n (%), or range, as appropriate. Treatment adherence was calculated as the number of completed prescribed rehabilitation sessions divided by the number of prescribed sessions ×100%. Good adherence was defined as completion of ≥80% of prescribed sessions. *Follow-up timing variables were calculated among participants with an evaluable follow-up assessment (conventional active rehabilitation comparator, n=144; multimodal rehabilitation, n=156). *P values* for continuous variables were obtained using independent-samples t tests or Mann–Whitney U tests, as appropriate, and categorical variables were compared using Pearson's χ² test or Fisher's exact test, as appropriate.

**Supplementary Table S4. Detailed Description of Rehabilitation Strategies According to the TIDieR Framework**

| **Item** | **conventional active rehabilitation comparator** | **Multimodal pelvic floor rehabilitation** |
| --- | --- | --- |
| Provider | Rehabilitation physician + trained rehabilitation therapist | Rehabilitation physician + trained pelvic floor rehabilitation therapist |
| Setting | Outpatient assessment + home training | Supervised outpatient rehabilitation + home training |
| Equipment | Not used | PHENIX USB4 neuromuscular stimulation therapy device (ELECTRONIC CONCEPT LIGNON INNOVATION, Montpellier, France) |
| Probe | Not used | Combined electrical stimulation and biofeedback intravaginal probe; the same probe was used for both treatment modes |
| General education | Postpartum education, posture, activity, diet/lifestyle guidance | Same as the conventional active rehabilitation comparator group |
| PFMT type | Basic home-based PFMT | Structured, supervised and progressive PFMT |
| PFMT slow contractions | 10 repetitions × 3 sets/day | 10 repetitions × 3 sets/session |
| Contraction duration | 5 s | 6–8 s |
| Relaxation duration | 5 s | 6–8 s |
| Rapid contractions | 10/set | 10/set |
| Home PFMT | 3 sets/day | 2 sets/day in addition to supervised treatment |
| Biofeedback | None | Surface EMG biofeedback prescription selected on the PHENIX USB4 device using the combined intravaginal probe |
| Biofeedback duration | — | 20 min/session |
| Biofeedback frequency | — | 2 sessions/week |
| Electrical stimulation | None | Low-frequency electrical stimulation prescription selected on the same PHENIX USB4 device using the same combined intravaginal probe |
| Frequency | — | 35 Hz |
| Pulse width | — | 300 μs |
| Duty cycle | — | 5 s on / 10 s off |
| Intensity | — | Individually titrated, approximately 10–30 mA |
| Stimulation duration | — | 15 min/session |
| Stimulation frequency | — | 2 sessions/week |
| Core training | General posture/activity advice | Transversus abdominis activation, pelvic control, bridge exercise, breathing coordination |
| Core training dose | — | 2 sets × 10–12 repetitions; ~20 min/session |
| Constipation management | General dietary/lifestyle advice | Structured defecation behavior training + diaphragmatic breathing + pelvic floor relaxation |
| Planned duration | 4–8 weeks | 4–8 weeks |
| Typical supervised frequency | ~1 reassessment/week | ~2 sessions/week |
| Actual sessions received | 5.0 (4.0–6.0) | 12.0 (9.0–14.0) |
| Treatment completion | 158/170 (92.9%) | 172/182 (94.5%) |
| Adherence definition | Completed prescribed sessions / prescribed sessions ×100% | Same |
| Mean treatment adherence, % | 89.6±9.8% | 90.8±9.1% |
| Adverse-event monitoring | Retrospective record review | Retrospective record review |

**Supplementary Table S5. Treatment-Related Adverse Events According to Rehabilitation Strategy**

| **Treatment-related adverse event** | **conventional active rehabilitation comparator (n=170)** | **Multimodal rehabilitation (n=182)** |
| --- | --- | --- |
| Transient pelvic/perineal discomfort | 2 (1.2) | 3 (1.6) |
| Transient muscle soreness | 3 (1.8) | 4 (2.2) |
| Mild vaginal irritation | 0 (0.0) | 2 (1.1) |
| Serious treatment-related adverse event | 0 (0.0) | 0 (0.0) |
| Discontinuation due to intolerance/adverse event | 1 (0.6) | 1 (0.5) |

**Note:** Data are presented as n (%). Adverse events were identified retrospectively from rehabilitation records and follow-up documentation and were not collected using a prospective standardized adverse-event surveillance protocol. Therefore, the reported frequencies should be interpreted as documented events rather than comprehensive estimates of adverse-event incidence.

**Supplementary Table S6. Sensitivity Analyses Using Alternative PAC-SYM Response Thresholds**

| **PAC-SYM response definition** | **Conventional** | **Multimodal** | **Unadjusted *P*** | **Sensitivity Model 1 *OR (95% CI)*** | ***P*** | **Sensitivity Model 2 *OR (95% CI)*** | ***P*** |
| --- | --- | --- | --- | --- | --- | --- | --- |
| Absolute reduction ≥0.60 points | 37 (25.7) | 136 (87.2) | <0.001 | 21.48 (11.17–41.30) | <0.001 | 7.49 (1.99–28.23) | 0.003 |
| Absolute reduction ≥0.75 points | 15 (10.4) | 130 (83.3) | <0.001 | 57.61 (26.00–127.65) | <0.001 | 28.87 (6.89–121.06) | <0.001 |
| Relative reduction ≥30% | 46 (31.9) | 143 (91.7) | <0.001 | 34.56 (15.78–75.66) | <0.001 | 8.20 (1.82–36.98) | 0.006 |

**Note:** The ≥0.60-point absolute reduction was used as the main supportive PAC-SYM responder threshold. An absolute reduction of ≥0.75 points was examined as a more conservative sensitivity threshold, whereas the ≥30% relative reduction represented the threshold used in the original analysis and was retained only for sensitivity analysis. Sensitivity Model 1 adjusted for clinically selected pre-treatment covariates defined for the present retrospective analysis; Sensitivity Model 2 additionally adjusted for actual treatment duration, number of treatment sessions, and treatment adherence.

**Supplementary Table S7. Diagnostics and Outcome Estimates From the Inverse-Probability-of-Follow-up-Weighted Analysis**

**Panel A. Weight diagnostics**

| **Diagnostic measure** | **Value** |
| --- | --- |
| Outcome-evaluable participants, n | 300 |
| Weight truncation | 1st and 99th percentiles |
| Truncated stabilized weight range | 0.884–1.328 |
| Mean stabilized weight | 0.998 |
| Effective sample size | 297.6 |
| Maximum absolute SMD before weighting | 0.0327 |
| Maximum absolute SMD after weighting | 0.0039 |
| Covariates with absolute SMD >0.10 after weighting, n | 0 |

**Panel B. IPCW-weighted outcome models**

| **Outcome** | **Adjusted *β (95% CI)*** | ***P value*** |
| --- | --- | --- |
| Follow-up NRS | −1.59 (−1.78 to −1.39) | <0.001 |
| Follow-up ODI | −9.63 (−10.52 to −8.73) | <0.001 |
| Follow-up PAC-SYM | −0.62 (−0.69 to −0.55) | <0.001 |
| Weekly bowel-movement frequency | 1.55 (1.41 to 1.70) | <0.001 |

**Note:** Stabilized inverse-probability-of-follow-up weights were calculated using a numerator model containing rehabilitation strategy and a denominator model containing rehabilitation strategy and clinically selected baseline covariates. Weights were truncated at the 1st and 99th percentiles. Weighted outcome models included the same baseline adjustment variables as the primary analysis and used robust sandwich standard errors. SMD, standardized mean difference; IPCW, inverse probability of follow-up weighting; NRS, Numerical Rating Scale; ODI, Oswestry Disability Index; PAC-SYM, Patient Assessment of Constipation Symptoms.

**Supplementary Table S8. Landmark-Anchored Missing-Data Sensitivity Analyses**

**Panel A. Observed and MAR-imputed outcome distributions**

| **Outcome** | **Group** | **Observed, mean ± SD** | **Observed median (IQR)** | **MAR-imputed, mean ± SD** | **MAR-imputed median (IQR)** |
| --- | --- | --- | --- | --- | --- |
| NRS | Conventional | 4.07 ± 1.26 | 4.00 (3.00–5.00) | 3.90 ± 1.71 | 4.00 (3.00–5.00) |
| NRS | Multimodal | 2.76 ± 1.51 | 3.00 (2.00–4.00) | 2.64 ± 1.55 | 3.00 (1.00–4.00) |
| ODI | Conventional | 28.52 ± 8.92 | 28.70 (24.35–35.00) | 28.85 ± 9.03 | 28.00 (23.10–35.75) |
| ODI | Multimodal | 19.88 ± 9.04 | 19.25 (13.20–25.13) | 19.91 ± 8.96 | 19.40 (13.20–24.90) |
| PAC-SYM | Conventional | 1.41 ± 0.48 | 1.35 (1.08–1.72) | 1.26 ± 0.58 | 1.22 (0.84–1.65) |
| PAC-SYM | Multimodal | 0.96 ± 0.49 | 0.97 (0.63–1.29) | 1.24 ± 0.59 | 1.29 (0.81–1.64) |
| Weekly bowel frequency | Conventional | 2.62 ± 1.02 | 2.50 (2.00–3.00) | 2.74 ± 1.06 | 3.00 (2.00–4.00) |
| Weekly bowel frequency | Multimodal | 4.09 ± 1.11 | 4.00 (3.00–5.00) | 3.67 ± 1.21 | 4.00 (3.00–5.00) |

**Panel B. Delta-adjustment sensitivity analysis**

| **Outcome** | **Scenario** | **Delta applied to missing multimodal outcomes** | **Adjusted**  ***β (95% CI)*** | ***P value*** |
| --- | --- | --- | --- | --- |
| NRS | MAR landmark | 0 | −1.47 (−1.69 to −1.26) | <0.001 |
| NRS | Mild MNAR | 0.50 | −1.40 (−1.62 to −1.18) | <0.001 |
| NRS | Moderate MNAR | 1 | −1.33 (−1.56 to −1.10) | <0.001 |
| NRS | Severe MNAR | 1.50 | −1.26 (−1.50 to −1.02) | <0.001 |
| ODI | MAR landmark | 0 | −9.59 (−10.56 to −8.62) | <0.001 |
| ODI | Mild MNAR | 3 | −9.16 (−10.16 to −8.17) | <0.001 |
| ODI | Moderate MNAR | 5 | −8.88 (−9.90 to −7.85) | <0.001 |
| ODI | Severe MNAR | 10 | −8.17 (−9.32 to −7.02) | <0.001 |
| PAC-SYM | MAR landmark | 0 | −0.58 (−0.66 to −0.51) | <0.001 |
| PAC-SYM | Mild MNAR | 0.15 | −0.56 (−0.64 to −0.49) | <0.001 |
| PAC-SYM | Moderate MNAR | 0.30 | −0.54 (−0.62 to −0.46) | <0.001 |
| PAC-SYM | Severe MNAR | 0.50 | −0.51 (−0.60 to −0.43) | <0.001 |
| Weekly bowel frequency | MAR landmark | 0 | 1.46 (1.31 to 1.62) | <0.001 |
| Weekly bowel frequency | Mild MNAR | −0.50 | 1.39 (1.23 to 1.55) | <0.001 |
| Weekly bowel frequency | Moderate MNAR | −1.00 | 1.32 (1.15 to 1.49) | <0.001 |
| Weekly bowel frequency | Severe MNAR | −1.50 | 1.25 (1.07 to 1.43) | <0.001 |

**Note:** For participants without evaluable follow-up, the assessment time was anchored at 56 days after baseline. Missing outcomes were imputed using predictive mean matching with 50 imputations. The delta-adjustment analysis progressively shifted imputed outcomes among participants in the multimodal rehabilitation group in a direction unfavorable to multimodal rehabilitation. Positive delta values indicate higher (worse) NRS, ODI, or PAC-SYM scores; negative delta values indicate lower weekly bowel-movement frequency. Estimates were pooled using Rubin's rules. MAR, missing at random; MNAR, missing not at random.

**Supplementary Table S9. Baseline Covariate Balance in the Propensity Score-Matched Outcome-Available Subset**

| **Variable** | **conventional active rehabilitation comparator (n=102)** | **Multimodal rehabilitation (n=104)** | **SMD** |
| --- | --- | --- | --- |
| Age, years | 31.27 ± 4.66 | 31.04 ± 4.26 | 0.053 |
| BMI, kg/m² | 23.73 ± 2.65 | 23.67 ± 2.84 | 0.022 |
| Postpartum time, weeks | 14.22 ± 5.82 | 14.43 ± 6.02 | 0.037 |
| Mode of delivery |  |  | 0.007 |
| └ Cesarean section | 33 (32.4) | 34 (32.7) |  |
| └ Vaginal delivery | 69 (67.6) | 70 (67.3) |  |
| Parity |  |  | 0.079 |
| └ 1 | 58 (56.9) | 59 (56.7) |  |
| └ 2 | 33 (32.4) | 36 (34.6) |  |
| └ ≥3 | 11 (10.8) | 9 (8.7) |  |
| Neonatal birth weight, g | 3318.82 ± 437.86 | 3290.19 ± 388.48 | 0.069 |
| Breastfeeding |  |  | 0.033 |
| └ No | 26 (25.5) | 28 (26.9) |  |
| └ Yes | 76 (74.5) | 76 (73.1) |  |
| Episiotomy |  |  | 0.011 |
| └ No | 78 (76.5) | 80 (76.9) |  |
| └ Yes | 24 (23.5) | 24 (23.1) |  |
| Degree of perineal laceration |  |  | 0.044 |
| └ Grade 0 | 58 (56.9) | 61 (58.7) |  |
| └ Grade 1 | 31 (30.4) | 31 (29.8) |  |
| └ Grade 2 | 12 (11.8) | 11 (10.6) |  |
| └ Grade 3 | 1 (1.0) | 1 (1.0) |  |
| Baseline NRS pain score | 5.14 ± 1.08 | 5.23 ± 1.30 | 0.078 |
| Baseline ODI score | 34.16 ± 7.92 | 34.97 ± 9.02 | 0.095 |
| Baseline PAC-SYM score | 1.86 ± 0.40 | 1.86 ± 0.43 | 0.008 |
| Baseline weekly defecation frequency | 2.21 ± 0.88 | 2.16 ± 0.87 | 0.048 |
| Baseline Bristol Stool Form Scale score | 2.61 ± 0.90 | 2.49 ± 0.85 | 0.134 |
| Baseline Oxford pelvic floor muscle strength grade | 2.70 ± 0.76 | 2.61 ± 0.69 | 0.125 |
| Baseline PFDI-20 score | 53.98 ± 9.40 | 54.19 ± 8.91 | 0.023 |
| Duration of low back pain, weeks | 9.34 ± 5.03 | 9.37 ± 4.90 | 0.004 |
| Duration of constipation, weeks | 8.23 ± 4.05 | 8.18 ± 4.20 | 0.010 |
| Analgesic use |  |  | 0.031 |
| └ No | 74 (72.5) | 74 (71.2) |  |
| └ Yes | 28 (27.5) | 30 (28.8) |  |
| Laxative use |  |  | 0.037 |
| └ No | 81 (79.4) | 81 (77.9) |  |
| └ Yes | 21 (20.6) | 23 (22.1) |  |

**Note:** Continuous variables are presented as mean ± standard deviation and categorical variables as n (%). This table reports baseline covariate balance after restriction of the full propensity score-matched treatment-initiation cohort to participants with evaluable follow-up outcomes (102 conventional and 104 multimodal participants). Covariate balance was assessed using absolute standardized mean differences (SMDs), with an absolute SMD <0.10 considered acceptable. Modest residual imbalance was observed for baseline Bristol Stool Form Scale score (SMD=0.134) and baseline Oxford pelvic floor muscle strength grade (SMD=0.125); all other measured baseline variables had absolute SMDs <0.10. Hypothesis-testing P values were not used to assess covariate balance. NRS, Numerical Rating Scale; ODI, Oswestry Disability Index; PAC-SYM, Patient Assessment of Constipation Symptoms; PFDI-20, Pelvic Floor Distress Inventory-20; BMI, body mass index.

**Supplementary Table S10. Diagnostics for the Inverse Probability of Treatment Weighting Analysis**

**Panel A. Propensity-score and treatment-weight diagnostics**

| **Diagnostic measure** | **Value** |
| --- | --- |
| Treatment-initiation cohort, n | 352 |
| conventional active rehabilitation comparator, n | 170 |
| Multimodal rehabilitation, n | 182 |
| Propensity-score range, conventional active rehabilitation comparator group | 0.077–0.890 |
| Propensity-score range, multimodal group | 0.095–0.903 |
| Weight truncation | 1st and 99th percentiles |
| Raw stabilized weight, minimum | 0.523 |
| Raw stabilized weight, 1st percentile | 0.554 |
| Raw stabilized weight, 5th percentile | 0.604 |
| Raw stabilized weight, median | 0.859 |
| Raw stabilized weight, 95th percentile | 1.812 |
| Raw stabilized weight, 99th percentile | 3.484 |
| Raw stabilized weight, maximum | 5.446 |
| Truncated stabilized weight range | 0.554–3.484 |
| Mean truncated stabilized weight | 0.998 |
| Effective weighted sample size, full cohort | 289.6 |
| Outcome-evaluable participants, n | 300 |
| Effective treatment-weighted outcome sample size | 248.4 |
| Empirical common-support interval | 0.095-0.890 |

Panel B. Covariate balance before and after IPTW

| **Baseline covariate** | **Before IPTW SMD** | **After IPTW SMD** |
| --- | --- | --- |
| Baseline PAC-SYM | 0.489 | 0.048 |
| Baseline PFDI-20 | 0.258 | 0.044 |
| Baseline Oxford strength | 0.226 | 0.049 |
| Neonatal birth weight | 0.2 | 0.001 |
| Episiotomy: Yes | 0.103 | 0.016 |
| Baseline ODI | 0.098 | 0.017 |
| Baseline Bristol Stool Form Scale | 0.098 | 0.027 |
| Duration of low back pain | 0.097 | 0.003 |
| Baseline NRS | 0.088 | 0.003 |
| Vaginal delivery | 0.084 | 0.003 |
| Baseline weekly defecation frequency | 0.08 | 0.007 |
| Age | 0.076 | 0.038 |
| Parity: 1 | 0.053 | 0.015 |
| Analgesic use: Yes | 0.049 | 0.013 |
| Parity: 2 | 0.048 | 0.011 |
| Breastfeeding: Yes | 0.034 | 0.002 |
| BMI | 0.031 | 0.008 |
| Laxative use: Yes | 0.023 | 0.003 |
| Postpartum time | 0.019 | 0.014 |
| Perineal laceration: Grade 3 | 0.016 | 0.005 |
| Perineal laceration: Grade 2 | 0.012 | 0.012 |
| Duration of constipation | 0.009 | 0.033 |
| Parity: ≥3 | 0.004 | 0.004 |
| Perineal laceration: Grade 0 | 0.002 | 0.01 |
| Perineal laceration: Grade 1 | 0.001 | 0.003 |

**Note:** Stabilized inverse probability of treatment weights were derived from the propensity-score model fitted in the full treatment-initiation cohort and were truncated at the 1st and 99th percentiles. Covariate balance was assessed using absolute standardized mean differences (SMDs), with an absolute SMD <0.10 considered acceptable. For categorical variables with more than two levels, indicator-level balance is presented to correspond with Supplementary Figure S4. The maximum absolute SMD decreased from 0.489 before IPTW to 0.049 after IPTW, and all measured baseline covariates or indicator levels met the balance criterion after weighting. The effective sample size was calculated as $\frac{{(\sum w)}^{2}}{{\sum w}^{2}}$. IPTW, inverse probability of treatment weighting; SMD, standardized mean difference; NRS, Numerical Rating Scale; ODI, Oswestry Disability Index; PAC-SYM, Patient Assessment of Constipation Symptoms; PFDI-20, Pelvic Floor Distress Inventory-20; BMI, body mass index.

**Supplementary Table S11. Distribution-Appropriate and Penalized Sensitivity Analyses**

**Panel A. Distribution-appropriate sensitivity analyses for continuous outcomes**

| **Outcome** | **Sensitivity model** | **Effect estimate *(95% CI)*** | ***P value*** |
| --- | --- | --- | --- |
| Follow-up weekly bowel-movement frequency | Poisson regression with robust SE | IRR=1.59 (1.51–1.67) | <0.001 |
| Follow-up PAC-SYM score | Beta regression | Adjusted marginal difference=−0.66 (−0.74 to −0.59) | <0.001 |

Pearson dispersion statistic for the Poisson model=0.135.

**Panel B. Firth penalized logistic regression for supportive responder outcomes**

| Outcome | Conventional logistic *OR (95% CI)* | Firth penalized  *OR (95% CI)* | *P value* |
| --- | --- | --- | --- |
| Pain responder | 33.33 (15.70–70.75) | 26.06 (13.27–55.53) | <0.001 |
| PAC-SYM MID responder | 21.48 (11.17–41.30) | 17.53 (9.64–33.50) | <0.001 |
| Bowel-frequency improvement ≥2/week | 176.84 (53.72–582.15) | 105.08 (40.24–353.42) | <0.001 |
| Dual pain + PAC-SYM MID response | 55.01 (24.73–122.37) | 39.80 (19.75–87.81) | <0.001 |

Note: The Poisson and beta-regression sensitivity models used the same clinically selected baseline adjustment set defined for the present analysis as the corresponding primary continuous-outcome models. The beta-regression marginal contrast was transformed back to the original 0–4 PAC-SYM scale. Firth penalized logistic regression used the same clinically selected pre-treatment covariates defined for the present retrospective analysis as Sensitivity Model 1 and was performed to assess the potential influence of sparse data and near-separation. Conventional logistic odds ratios are shown for comparison. IRR, incidence rate ratio; *OR*, odds ratio; PAC-SYM, Patient Assessment of Constipation Symptoms; MID, minimal important difference.

**Supplementary Table S12. Exploratory Within-Multimodal Treatment-Exposure Analysis**

| **Exposure** | **Follow-up outcome** | **Adjusted *β (95% CI)*** | ***P value*** |
| --- | --- | --- | --- |
| Each additional treatment session | NRS | −0.083 (−0.133 to −0.034) | 0.001 |
| Each additional treatment session | ODI | −0.086 (−0.334 to 0.162) | 0.496 |
| Each additional treatment session | PAC-SYM | −0.025 (−0.043 to −0.006) | 0.009 |
| Each additional treatment session | Weekly bowel-movement frequency | 0.056 (0.019 to 0.093) | 0.003 |
| Each additional treatment week | NRS | −0.307 (−0.459 to −0.155) | <0.001 |
| Each additional treatment week | ODI | −0.483 (−1.219 to 0.253) | 0.198 |
| Each additional treatment week | PAC-SYM | −0.073 (−0.138 to −0.008) | 0.029 |
| Each additional treatment week | Weekly bowel-movement frequency | 0.140 (0.015 to 0.265) | 0.028 |

**Note:** Analyses were restricted to the 156 participants in the multimodal rehabilitation group with evaluable follow-up outcomes. Session number and actual treatment duration were examined in separate multivariable models because they were correlated measures of treatment exposure (Pearson r=0.688). Models were adjusted for age, BMI, postpartum time, mode of delivery, parity, baseline NRS, baseline ODI, baseline PAC-SYM, baseline weekly bowel-movement frequency, baseline Oxford pelvic floor muscle strength grade, analgesic use, laxative use, and baseline-to-follow-up interval. HC3 heteroscedasticity-consistent standard errors were used. Negative β coefficients for NRS, ODI, and PAC-SYM and positive β coefficients for weekly bowel-movement frequency indicate more favorable follow-up outcomes with greater treatment exposure. Because treatment exposure was determined after treatment initiation, these analyses were exploratory and should not be interpreted as causal dose-response estimates.

**Supplementary Figure S1. Baseline Distribution Before and After Inverse-Probability-of-Follow-up Weighting.**

**Supplementary Figure S2. Distribution of Truncated Stabilized Inverse-Probability-of-Follow-up Weights.**

**Supplementary Figure S3. Landmark-Anchored Delta Sensitivity Analysis Under Increasing Departures From MAR.**

**Supplementary Figure S4. Propensity-Score Overlap and Covariate Balance Before and After Inverse Probability of Treatment Weighting.**
